# Multiple Sclerosis-Associated Gut Microbiome in the Israeli Diverse Populations: Associations with Ethnicity, Gender, Disability Status, Vitamin D Levels, and Mediterranean Diet

**DOI:** 10.3390/ijms241915024

**Published:** 2023-10-09

**Authors:** Zehavit Nitzan, Elsebeth Staun-Ram, Anat Volkowich, Ariel Miller

**Affiliations:** 1 Rappaport Faculty of Medicine, Technion-Israel Institute of Technology, Haifa 3109601, Israelelsebeth@technion.ac.il (E.S.-R.); 2 Neuroimmunology Unit & Multiple Sclerosis Center, Lady Davis Carmel Medical Center, Haifa 3436212, Israel; 3 Department of Neurology, Lady Davis Carmel Medical Center, Haifa 3436212, Israel

**Keywords:** diet, dysbiosis, EDSS, ethnicity, gender, microbiome, multiple sclerosis, nutrient intake

## Abstract

Microbiome dysbiosis is increasingly being recognized as implicated in immune-mediated disorders including multiple sclerosis (MS). The microbiome is modulated by genetic and environmental factors including lifestyle, diet, and drug intake. This study aimed to characterize the MS-associated gut microbiome in the Israeli populations and to identify associations with demographic, dietary, and clinical features. The microbiota from 57 treatment-naive patients with MS (PwMS) and 43 age- and gender-matched healthy controls (HCs) was sequenced and abundance compared. Associations between differential microbes with demographic or clinical characteristics, as well as diet and nutrient intake, were assessed. While there was no difference in α- or β-diversity of the microbiome, we identified 40 microbes from different taxonomic levels that differ in abundance between PwMS and HCs, including *Barnesiella*, *Collinsella*, *Egerthella*, *Mitsuokella*, *Olsenella Romboutsia*, and *Succinivibrio*, all enhanced in PwMS, while several members of *Lacnospira* were reduced. Additional MS-differential microbes specific to ethnicity were identified. Several MS-specific microbial patterns were associated with gender, vitamin D level, Mediterranean diet, nutrient intake, or disability status. Thus, PwMS have altered microbiota composition, with distinctive patterns related to geographic locations and population. Microbiome dysbiosis seem to be implicated in disease progression, gender-related differences, and vitamin D-mediated immunological effects recognized in MS. Dietary interventions may be beneficial in restoring a “healthy microbiota” as part of applying comprehensive personalized therapeutic strategies for PwMS.

## 1. Introduction

The disease etiology of multiple sclerosis (MS) appears to include interactions between genetics, lifestyle, and environmental risk factors, which may also affect disease activity and progression. The main demographic and environmental factors associated with MS include obesity, Epstein–Barr virus infection, vitamin D deficiency, and smoking [1]. Recent accumulating data suggest that the gut microbiome may also contribute to MS’s pathogenesis and clinical phenotype. The gut microbiome plays a major role in health and disease and is involved in the development and activation of the human immune system, protection from infectious pathogens, food digestion, energy homeostasis, vitamin production, and intestine barrier integrity [2,3]. Furthermore, the microbiome is part of a cross-talk between the gut and the brain; the gut–brain axis, facilitated though bidirectional pathways, including microbial-secreted metabolites and neurotransmitters; afferent and efferent nerves; and the hypothalamic–pituitary–adrenal axis [3,4].

The link between gut dysbiosis and autoimmunity may include molecular mimicry between microbe-antigens and host auto-antigens, bystander inflammatory T cell activation, and imbalance between regulatory and effector T cells in an inflammatory milieu [5,6]. In an animal model of spontaneous experimental autoimmune encephalomyelitis (EAE), a model of relapsing–remitting MS, mice housed in a germ-free environment were refractory from EAE but developed disease when recolonized with conventional commensal microbiota [7]. Moreover, germ-free mice colonized with fecal samples from MS patients developed a higher incidence of EAE and reduced proportions of regulatory T cells (Tregs) than mice colonized with fecal samples from healthy individuals, indicative that the MS gut microbiome contains factors that promote disease [8,9].

The gut microbiome is influenced by numerous factors including host genetics, age, geographical location, medications, lifestyle, and diet [10], and characterization of a disease-associated microbiome is, therefore, challenging. Although several studies have described an MS-associated microbiome, these have showed relatively low repetition and only few commonalities across studies. The Israeli population is comprised of relatively defined endogamous ethnicities, with cultural and traditional eating habits, mostly associated with a Mediterranean diet. We aimed, in this study, to characterize the MS-associated gut microbiome in the Israeli populations, including solely treatment-naïve patients, and to identify associations between the microbiome and nutritional data, vitamin D serum level, and disease state.

## 2. Results

### 2.1. Subject Demographics

We recruited 57 treatment-naïve, relapsing–remitting MS (RRMS) patients and 43 HCs belonging to the two major ethnicities in Israel: 63 Jewish and 37 Arab participants (Table 1). There was no statistically significant difference between PwMS and HCs in age, BMI, or proportions of gender, smoking, vegetarian, or ethnicity. PwMS had a relatively short disease duration (2.6 ± 0.6 months, median 0.1 month) and accordingly low EDSS (Expanded Disability Status Scale) (1.6 ± 0.1, [range 0–4]), with an annual relapse rate of 0.85 ± 0.07, reflecting the proximity to disease onset and diagnosis, which is likely to involve at least one recent clinical episode of disease activity (Table 1).

### 2.2. General Microbiome Composition

**Figure 1:**
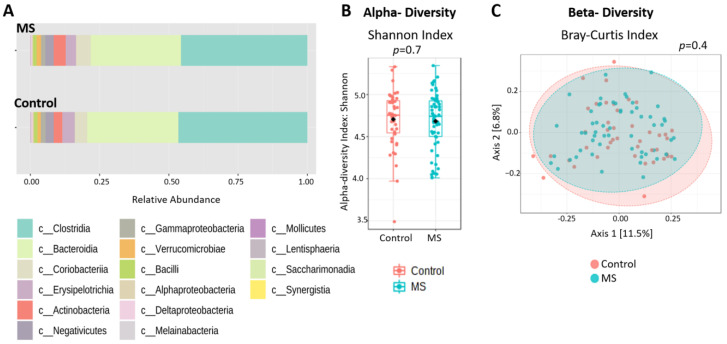
**General microbiome composition.** (**A**): Relative composition at the class level. (**B**): Alpha-diversity between MS and HC calculated by Shannon index at the OTU level. (**C**): Beta-diversity between MS and HC calculated by Bray–Curtis dissimilarity at the OTU level. MS—multiple sclerosis.

In total, 1295 OTUs were available for analysis after filtration. The general microbiome composition was similar between PwMS and HCs (shown in Figure 1A for the class level). There was no significant difference between the groups in either α-diversity (Shannon index) or β-diversity (Bray–Curtis dissimilarity) (Figure 1B,C). There was also no difference in α- or β-diversity when analyzing all samples according to gender, age (3 age-groups), ethnicity (Jews and Arabs), smoking status, and adherence to a Mediterranean diet (MDS low, 1–6 points; intermediate, 7–11 points; high adherence, 12–17 points) (Figure S1). β-diversity differed according to BMI (four groups: underweight (<18.5), healthy weight (18.5–24.9), overweight (25–29.9), and obesity (>30)) (*p* = 0.031), which is well known to affect microbiome composition [11,12] (Figure S1), and thus was adjusted for intra-group microbiome assessments where relevant.

### 2.3. Differentially Abundant Taxa

**Figure 2:**
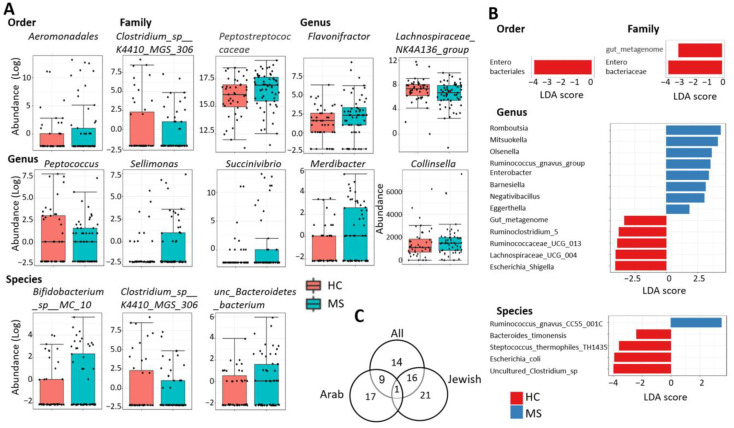
**Differential abundant taxa between PwMS and HCs.** (**A**)—Representative graphs of taxa with significant difference in relative abundance, as determined by at least two out of three tools (DESeq2, MetagenomeSeq, and EdgeR) at different taxonomic levels (FDR < 0.1). Each black dot represents the relative abundance of a participant sample. (**B**)—Linear discriminant analysis (LDA) score of significant different taxa as determined by LDA effect size (LefSE) between PwMS and HCs at different taxonomic levels (*p* < 0.05). (**C**)—Venn diagram presenting the overlap of differentially abundant taxa identified in the analysis of all samples, Jewish participants or Arab participants. HC—healthy control, MS—multiple sclerosis.

With several tools available for testing differential abundance in the microbiome and little consensus regarding the best practice, we adopted a cautious approach as recommended [13], and employed four different methods to identify the most consistently altered taxa across tests. Moreover, we used as default a low filtration approach to include taxa with low counts, e.g., OTUs with ≥4 counts in ≥10% of samples. Since statistical adjustment for multiple hypothesis testing (by false discovery rate, FDR) may increase the non-discovery rate of differential taxa with small effect size, we performed, as a complement, similar differential analyses after a medium filter of ≥4 counts in at least 20% of samples + ≥20% variance, and a strict filter of ≥5 counts in at least 50% of samples + ≥20% variance. Taxa with differential relative abundance identified by at least two out of three tools (DESeq2, MetagenomeSeq, and EdgeR) at FDR < 0.1, or taxa discriminating between PwMS and HCs according to LEfSe at *p*-value < 0.5 and LDA > 1.8 are listed in Table 2, and representative graphs are presented in Figure 2A,B. Forty taxa with differential abundance between PwMS and HCs were identified, including two orders, five families, 23 genera, and 10 species (Table 2). Of these, 25 taxa were differentially abundant in at least two out of three of the differential abundance methods; 21 taxa discriminated between MS and HC according to LEfSe (linear discriminant analysis effective size method), while 8 taxa were significant in both at least two out of three differential abundance methods as well as by LEfSe. The latter included the unspecified family *gut metagenome*, the genera *gut metagenome*, *Mitsuokella*, *Negativibacillus*, *Olsenella*, *Ruminococcaceae UCG-013*, and the species *Ruminococcus gnavus CC55 001C* and *uncultured Clostridium* sp. *1*; those taxa must be regarded as the most robust differential taxa, significant across three or four different tests.

Since the microbiome is affected, among other factors, by populations, and our cohort included participants from the two major ethnic populations in Israel (Jewish and Arab), a sub-analysis was performed separately for each ethnicity. In total, 21 differentially abundant taxa were identified uniquely in the Jewish cohort, 17 were identified uniquely in the Arab cohort, while 17 taxa overlapped between the full cohort and the Jewish cohort and 10 overlapped between the full cohort and the Arab cohort, likely reflecting the larger size of the Jewish cohort. Fourteen taxa were only significant in the full cohort, while one differential taxa was shared between all analyses: the genus *Mitsuokella*. (Table S1A,B, Figure 2C).

### 2.4. Associations between MS-Differentially Abundant Microbiota and Clinical Factors

**Figure 3:**
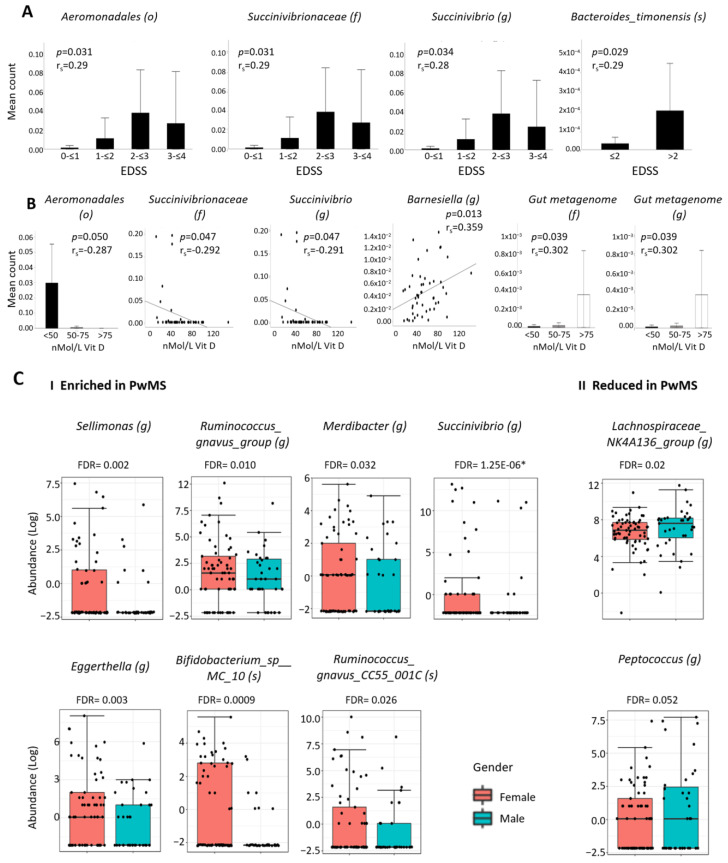
**Associations between MS-differentially abundant microbiota, EDSS, vitamin D, and gender.** (**A**)—Representative graphs of MS-differentially abundant taxa, which significantly correlates with EDSS (Spearman correlation, adjusted for BMI). (**B**)—Representative graphs of MS-differentially abundant taxa, which significantly correlates with serum vitamin D level shown as continuous or divided into 3 groups: <50 nMol/L—vitamin D insufficiency, 50–75 nMol/L—vitamin D mild deficiency, >75 nMol/L—vitamin D sufficient (Spearman correlation, adjusted for BMI). (**C**)—Representative graphs of MS-differentially abundant taxa enriched in PwMS (**I**) or reduced in PwMS (**II**), which differ significantly between females and males. Figure presents FDR calculated from EdgeR, * FDR from DeSeq2. Each black dot represents the relative abundance of a participant sample.

To identify correlations between abundance of the 40 MS-differentially abundant taxa and clinical presentation, we divided patients according to their EDSS at sample collection. Since patients were newly diagnosed, EDSS was in all cases ≤ 4. Patients were divided into two or four groups of EDSS. Despite the low EDSS, we identified five taxa enriched in PwMS—which significantly correlated positively with EDSS—of which three remained significant when controlled for BMI: the order *Aeromonodales* (*p* = 0.031,r = 0.29), the family *Succinivibrionaceae* (*p* = 0.031, r = 0.29), and the genus *Succinivibrio* (*p* = 0.034, r = 0.28). The genera *Olsenella* and *Lachnospiracea* correlated significantly when unadjusted for BMI (*p* = 0.029, r = 0.29; *p* = 0.046, r = 0.27, respectively). One species, *Bacteroides timonensis*, reduced in PwMS, also correlated with EDSS, after BMI adjustment (*p* = 0.029, r = 0.29) (Figure 3A).

Next, we examined whether the 40 MS-differentially abundant taxa correlated with vitamin D serum levels of the patients. Patients were divided into groups based upon the World Medical Association (WMA) criteria [14]: group1: <50 nMol/L, insufficiency; group2: 50–75 nMol/L, mild deficiency; group3: 75–100 nMol/L, sufficient vitamin D. Six taxa correlated significantly with vitamin D, after correction of BMI: three MS-enriched taxa from the same taxonomic branch, namely, the genus *Succinivibrio* (*p* = 0.047, r = −0.29), the family *Succinivibrionaceae* (*p* = 0.047. r = −0.29), and the order *Aeromonadales* (*p* = 0.050, r = −0.29) correlated negatively with vitamin D, while two MS-reduced taxa correlated positively with vitamin D, namely, the unspecified family *Gut metagenome* (*p* = 0.039, r = 0.3) and genus *Gut metagenome* (*p* = 0.039, r = 0.3), suggestive that achievement of a sufficient vitamin D level may contribute to modulate altered microbiota levels towards a healthy microbiome (Figure 3B). Additionally, the MS-enriched genus *Barnesiella* correlated positively with vitamin D, while the MS-enriched genus *Mitsoukella* correlated negatively with vitamin D, when unadjusted for BMI (*p* = 0.026, r = −0.322).

### 2.5. Differential Abundant Taxa and Gender

With the higher prevalence of MS in women than in men [15], we analyzed whether the abundance of the differential taxi differed between genders. Out of the 40 MS-differential taxa, 14 differed significantly in relative abundance between females and males. Of these, seven taxa enriched in PwMS were more abundant in females, namely, the genera *Sellimonas* (FDR = 0.002), *Ruminococcus_gnavus_group* (FDR = 0.010), *Merdibacter* (FDR = 0.032), *Succinivibrio* (FDR = 1.25 × 10^−6^), *Eggerthella* (FDR = 0.003), and the species *Bifidobacterium_sp__MC_10* (FDR = 0.0009) and *Ruminococcus_gnavus_CC55_001C* (FDR = 0.026), whereas three taxa reduced in PwMS were more abundant in males, namely, the genera *Lachnospiraceae_ NK4A136_group* (FDR = 0.02), *Peptococcus* (FDR = 0.052), and *Azospirillum* sp. *47-25* (FDR = 0.007 (the latter not presented due to very low count)) (Figure 3C). Since there was no significant difference in the female/male ratio between the MS and the healthy cohort in our study, the finding of 10 taxa with significant correlations both with an MS and with a female phenotype suggests a possible causative association between (female) gender, microbiota, and MS.

### 2.6. Differential Abundant Taxa and Dietary Data

**Figure 4:**
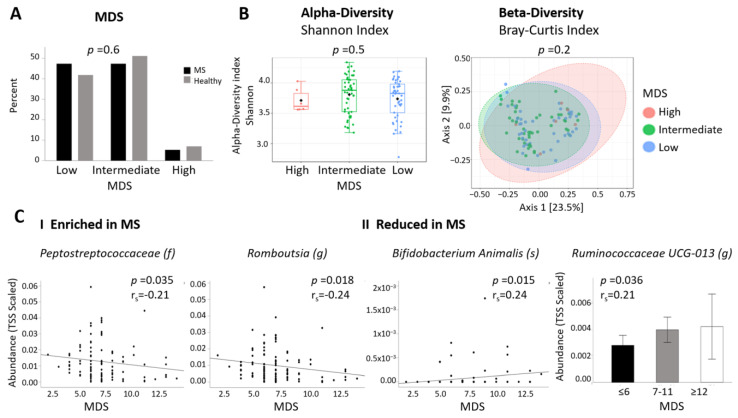
**Correlations between MS-differentially abundant microbiota and dietary data.** (**A**)—The distribution of adherence to a Mediterranean diet (MDS) in PwMS and HCs (Kruskal–Wallis test). (**B**)—Alpha-diversity Shannon index and beta-diversity Bray–Curtis dissimilarity according to high, low, or intermediate MDS (OTU level). (**C**)—Representative graphs of differentially abundant taxa, enriched in PwMS (**I**) or reduced in PwMS (**II**) which significantly correlates with MDS (Spearman correlation). MDS shown as continuous or divided into 3 groups (MDS low (1–6 points), intermediate (7–11 points), high (2–17 points). MDS—Mediterranean diet score.

Dietary information from participants included a Mediterranean diet score (MDS), an indicator of adherence to a Mediterranean diet, and the mean daily intake of energy as well as of 74 various nutrients, as analyzed from an FFQ, using questionnaires specifically adapted to the Israeli population. The MDS questionnaire provides a score from 0 to 17 points according to the reported intake of specified food. Participants were divided into three groups: low, intermediate, and high Mediterranean diet adherence. Most participants had intermediate or low adherence to a Mediterranean diet (94.7% of PwMS, 93.1% of controls) with no significant difference between cohorts (Table 1, Figure 4A). There was also no difference in MDS between the two major ethnicities (Jewish versus Arab, *p* = 0.67), or between PwMS and HCs in a sub-analysis within each of the two ethnicities (Jewish, *p* = 0.39 and Arab, *p* = 0.47, respectively). Analysis of the microbiome composition according to MDS categories showed no difference in α-diversity or β-diversity (Figure 4B). Next, we analyzed whether MDS was associated with the abundance of the 40 taxa that differed between PwMS and HCs. Two taxa enriched in PwMS correlated negatively with MDS, namely, the *Peptostreptococcaceae* family (*p* = 0.035, r = −0.21) and the *Romboutsia* genus (*p* = 0.018, r = −0.24) belonging to the same family, while two taxa reduced in MS, the genus *Ruminococcaceae UCG-013* (*p* = 0.036, r = 0.21) and the species *Bifidobacterium Animalis* (*p* = 0.015, rho = 0.24), correlated positively with MDS (Figure 4C). This suggests that improving adherence to a Mediterranean diet may potentially contribute to “repair” altered abundance of specific microbiota in MS.

**Figure 5:**
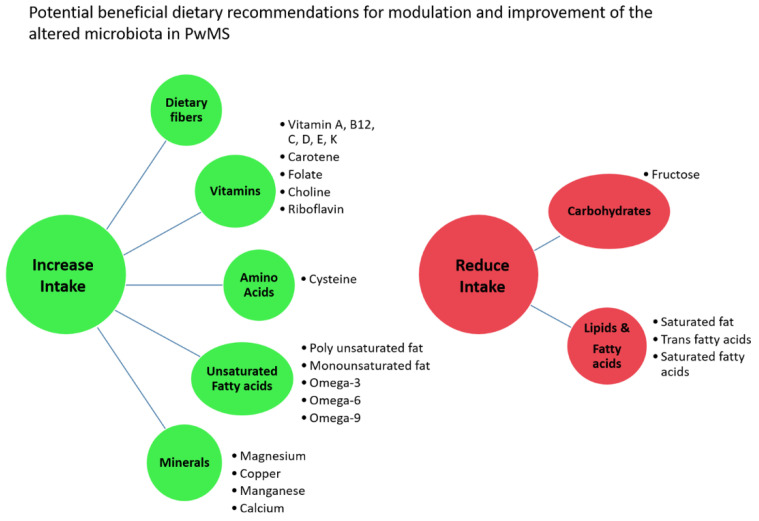
**Dietary recommendations.** Potential beneficial dietary recommendations for modulation and “repair” of altered microbiota abundance in PwMS, based upon correlations between nutrients and MS-differentially abundant taxa.

Analysis of the FFQ provides data on the estimated daily intake of 74 nutrients and energy. There was no difference in energy or nutrient intake between PwMS and HCs, besides alcohol intake, which was lower in PwMS (*p* = 0.032) (Table S2). Sub-analyses in Jewish or Arab participants separately or in female and male participants separately did not reveal additional differences in nutrient intake between PwMS and HCs (*p* = 0.039 in females, *p* > 0.05 for all other nutrients in females or males and in Jewish or Arab participants). Out of the 40 identified MS-differential taxa, significant correlations were found between the abundance of 25 taxa and 44 nutrients (Table S3). These included mostly vitamins and unsaturated fatty acids, but also amino acids, minerals, carbohydrates, lipids, saturated fatty acids, etc. Several correlations indicate that modification of the daily intake of specific nutrients could have a beneficial influence on the abundance of taxa altered in PwMS. For example, increased intake of various vitamins including vitamin B12, vitamin K, vitamin E, carotene, vitamin A, choline, and folate correlated negatively with the abundance of several microbiota enriched in PwMS, such as the genera *Eggerthella*, *Flavonifractor*, *Negativibacillus* and *Mitsuoke*, while vitamin C, D, and riboflavin (vitamin B2) correlated positively with genus *Lachnospiraceae UCG-004*, which was reduced in PwMS. Increased intake of minerals such as magnesium, copper, and manganese correlated negatively with the abundance of microbiota such as those of the *Peptostreptococcaceae* family or the genera *Romboutsia*, *Flavonifractor*, or *Negativibacillus*, all enriched in PwMS, while increased intake of the amino acid cysteine correlated positively with genus *Lachnospiraceae UCG-004*, which was reduced in PwMS. Higher intake of polyunsaturated fat, omega-3, omega-6, and omega-9 fatty acids, or Docosapentaenoic acid (DPA) correlated negatively with the abundance of PwMS-enriched microbiota such as the genera *Eggerthella*, *Flavonifractor*, or *Mitsuokella.* In contrast, trans fatty acids, saturated fat, and saturated fatty acids correlated positively with taxa enriched in PwMS, such as the species *Ruminococcus gnavus CC55_001C*, and correlated negatively with the abundance of taxa reduced in MS, such as those of the genus *Ruminococcaceae UCG-013* or the species *uncultured Clostridium* spp. and *Bacteroides timonensis*. A summary of 15 significant correlations, which support the concept that modifications of nutrient intake may “repair” altered abundance of specific taxa in PwMS, is presented in Table S4. Based upon these findings, in Figure 5, we present suggested dietary changes with the potential of beneficially modulating dysbiosis in PwMS and restoring a healthy microbiome. These are preliminary suggestions of focus for further research and exploration, presented with caution, since, in some cases, these nutrients also correlated oppositely with other altered microbiota, opposing “a repair” of dysbiosis.

### 2.7. Functional Pathways

**Figure 6:**
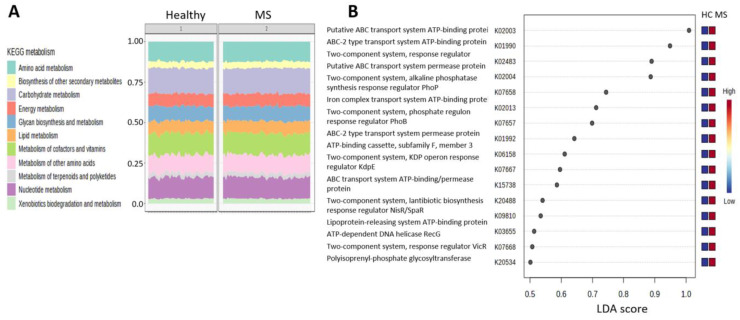
**Predicted functional pathways.** (**A**). Main KEGG metabolism pathways in the MS and healthy control cohorts (**B**). KOs discriminating between PwMS and HCs according to LEfSe at FDR < 0.1. KEGG—Kyoto Encyclopedia of Genes and Genomes, LEfSe: linear discriminant analysis effect size.

In order to explore the functional differences between the microbiome of PwMS and HCs, we used the Tax4Fun2 package in MicrobiomeAnalyst, which predicts functional profiles based upon the Kyota Encyclopedia of Genes and Genomes (KEGG) database [16,17]. The 1295 identified OTUs were transformed into 6277 KEGG orthologies (KOs), which were uploaded into the Shotgun Data Profiling module in MicrobiomeAnalyst for enrichment analysis and metabolic network mapping. Figure 6A shows the composition of KEGG metabolism pathways in both cohorts, the most abundant being amino acid metabolism, biosynthesis of other secondary metabolites, and carbohydrate metabolism. Differential abundance analysis (Kruskal–Wallis) revealed 485 KOs that differed between PwMS and HCs at FDR < 0.1, and these mapped significantly to nine KEGG pathway network maps, the highly significant top hit being biosynthesis of amino acids (FDR = 1.55^−19^), while other pathways included 2-Oxocarboxylic acid metabolism, Peptidoglycan biosynthesis, Pyrimidine metabolism, Alanine, aspartate, and glutamate metabolism, etc. (FDR < 0.05) (Table S5). Sixteen KOs, all enriched in PwMS compared to HCs, were identified as significant differential indicators between groups by LEfSe, but with a low effect size (LDA 0.5–1), (Figure 6B).

## 3. Discussion

The gut microbiota is affected by both genetic and environmental factors, thus, it may differ among populations. Most currently available reports on the microbiome in MS come from studies in the US, Europe, China, or Japan, with relatively low consistency in the identified microbiome profile across studies [18,19,20,21,22,23,24,25]. We aimed, in this study, to characterize the MS-associated microbiome in the Israeli population. The Israeli population is estimated at 9,656,000 residents (1.2023), comprised of 73.6% Jews, 21.1% Arabs, and 5.3% others [26]. The major Israeli populations, defined by their ancestral religious affiliation, mostly maintain endogamy within their communities; thus, they are considered to have relatively homogeneous genetic backgrounds [27,28,29]. We assessed differences in the microbiome in newly diagnosed, treatment-naïve PwMS compared to matched controls, eliminating confounding factors such as effects of disease modifying therapies (DMTs), disease duration, and progression. The overall microbiome composition was similar, with no difference in α-diversity or β-diversity, in line with most previous reports [6,30]. Analysis at the phylum to species level identified 40 taxa at different taxonomic levels, which significantly differed in relative abundance between PwMS and HCs, with 21 taxa enriched and 19 taxa reduced in PwMS. These taxa were identified adopting a cautious approach of using multiple available statistical methods, in order to identify taxa that are most robust across methods and, thus, are most likely to have biological relevance [13]. Some of the identified MS-differential taxa confirm reports from other geographical locations. The enrichment of *Eggerthella* in MS was also reported in a Japanese [24] and in a US study [20], while enrichment of *Flavonifractor* was also demonstrated by a Chinese [31] and a German group [32]. The enrichment of *Olsenella* supports a report from a Belgium study [33], while it was reduced in RRMS in a US study [34]. The enrichment of the *Peptostreptococcaceae* family and the related genus *Romboutsia* was reported, by a Chinese group, to be associated with five autoimmune diseases including MS [35]. The increase in *Collinsella* in PwMS confirms results from an Italian study [36], whereas, in contrast, *Collinsella* was reduced in MS patients in two US studies [22,23]. *Bifidobacterium animalis*, reduced in PwMS in our study, is a probiotic, which reduced the duration of clinical symptoms in EAE [37]. An unidentified species of the genus *Lachnospiraceae NK4A136*, a butyrate producer reduced in our MS cohort, reduced clinical relapses and MRI activity in a US study of pediatric MS [38]. In contrast, *Barnesiella*, enriched here in PwMS, was reduced in MS in two US studies [20,22], and *Tyzzerella 4*, reduced in our study, was increased in pediatric RRMS patients in a Canada–USA study [19]. Conflicts in identified differential taxa between studies can be a consequence of variations in methodology for DNA extraction, sequencing platforms, and statistical analysis. Differences in populations and geographic locations affect the microbiome and may also account for the relatively low consistency of findings across studies [10,39,40] as well as inclusions of patients receiving various DMTs, each with a profound effect on the microbiome [34,41], in contrast to treatment-naïve patients, as in our study. Additionally, our patients were recruited close to disease diagnosis, thus minimizing alterations that reflect disease progression and duration, accumulating comorbidities, etc. A single prior publication on the microbiome in Israeli PwMS conducted metagenomic sequencing and reported 23 species with differential abundance between MS and healthy participants [42], none of which overlapped the detected differential taxa in our study. There are several possible explanations for the lack of similarity: (1) their patient cohort was heterogeneous regarding disease types (clinically isolated syndrome, relapsing, progressive MS), patients in remission or during relapse, and treated with various DMTs; (2) they used rectal swab samples; (3) we used the 16S sequencing method, which has restricted ability to identify OTU at the level of species [43].

The sub-analyses performed according to ethnicity revealed differentially abundant taxa in PwMS unique for either the Jewish or the Arab participants, in addition to taxa overlapping the analysis of all samples, a finding that may possibly explain ethnicity-related clinical MS phenotypes among Israeli populations, as previously reported [29]. The genus *Mitsuokella* was enriched in PwMS in all analyses and was robust across the statistical tests. This microbe has not previously been reported in MS studies, but enrichment was found in autism spectrum disorder [44], prediabetic patients [45], and Behçet’s syndrome [46]. Among the taxa uniquely identified in the Jewish participants were the genera *Prevotella 9*, reduced in PwMS, and *Bilophila*, enriched in PwMS. *Bilophila* was previously found to be three times more abundant in children with MS [18]. Reduced abundance of *Prevotella* in PwMS has been reported in studies from Japan [24], US [23], and Italy [47] and *Prevotella* was enriched in patients on DMT compared to untreated patients [22]. *Prevotella* is a butyrate-producing genus, associated with anti-inflammatory properties, induction of Tregs, and intake of high-fiber diets [22], and the reduction in *Prevotella* in PwMS has been associated with high disease activity and increased levels of intestinal Th17 cells [47]. The identification of ethnicity-specific differential abundant taxa in PwMS indicates that population-specific microbiota patterns, affected by genetic and environmental factors, may influence a disease-associated microbiome and that ethnicity/populations should be considered in microbiome studies. We and others reported in previous studies that the prevalence rate (PR) in Israeli populations was lower in Arabs than in Jews [27,48]. Moreover, the PR was lower in Israeli Arabs and Jewish immigrants from Arab countries (Asia/Africa) than in native-born Jews of the same origin, which had a PR similar to European/American-origin Jews, suggesting environmental and lifestyle factors, rather than genetic factors, influence PR. Since environmental exposures such as climate and sun exposure are similar for all Israeli populations, due to residence within a small geographical location [49], other factors like diet, specific nutrient intake, and gut microbiota could possibly underlie the differences in PR. Whether the MS-differential microbes found specifically in the Jewish or the Arab cohort in this study may be related to the differential PR of MS between Arabs and Jews in Israel, and whether specific ethnicity-related nutrient intake correlates with these differences, remains to be explored. A study addressing the contribution of three ethnic groups in the US (Caucasian Americans, Hispanic Americans, and African Americans) to the MS microbiome also found no difference in α- or β-diversity. Differential abundant taxa between PwMS and controls were identified uniquely per ethnicity or shared between two, while only one taxa was shared between all three ethnicities [25].

Despite the short disease duration, we found three MS-enriched taxa from the same taxonomic branch, which correlated positively with EDSS both before and after controlling for BMI, while another two MS-enriched taxa correlated significantly only without BMI adjustment. In a US study, the abundance of four microbes, including *Collinsella aerofaciens* belonging to the genus *Collinsella* enriched in PwMS in our study, correlated positively with EDSS, while another microbe correlated negatively [12]. Another US group found correlations between several Clostridium species and EDSS in RRMS and PPMS patients, while butyrate producers such as the *Ruminococcus* species correlated negatively with EDSS [34]. These results imply that specific microbes may be associated with disease activity, progression, and degree of disability in MS. In pediatric RRMS patients, *Fusobacteria* depletion, enrichment of *Firmicutes*, and presence of *Euryarchaeota* (Archaea) were associated with a shorter time to relapse [50]. Eleven taxa belonging to *Firmacutes* were enriched in PwMS in our study. In pediatric-onset MS patients, five microbes were associated with disease activity, including two butyrate producers, an unidentified species in the *Lachnospiraceae NK4A136* group, and *Ruminococcaceae*, all of which were protective, while an unspecified member of *Coriobacteriales* was associated with increased risk of disease activity [38]. Interestingly, *Olsenella*, which correlated positively with EDSS in our study, belongs to *Coriobacteriales*, supporting an association with disease activity, while the *Lachnospiraceae NK4A136* group was reduced in our PwMS, supporting the finding that depletion of this microbe may contribute to disease activity.

Higher levels of vitamin D are associated with reduced risk of MS and clinical activity, and optimization of vitamin D levels by supplementation is commonly integrated in MS clinical practice [51,52,53]. Vitamin D has important anti-inflammatory and metabolic functions [51,52,54] and affects gastrointestinal motility by promoting intestinal calcium absorption and the integrity of the intestinal barrier, and vitamin D deficiency may lead to dysbiosis of the gut microbiota [55,56]. We identified three taxa enriched in PwMS, including those of the *Succinivibrionaceae* family, associated with pathogenesis of inflammatory diseases [46,57], which correlated negatively with vitamin D levels of the patients, while two unspecified *gut metagenome* taxa reduced in PwMS correlated positively with vitamin D. Thus, part of the beneficial effect of optimizing vitamin D levels in PwMS, may include the “repair” of altered taxa.

Gender differences in MS include a wide spectrum of aspects such as increased disease susceptibility and differences in disease progression and activity [58]. Women are more susceptible to MS than men, in an increasing ratio of ~3.5:1 [59]. This difference might be caused by gonadal hormones, where estrogens promote cell-mediated and humoral immunity, while androgens suppress immunity [60,61,62]; more robust immune responses in women than in men [60,61,63]; genetic differences such as the X chromosome [60,64]; and differences in exposure to environmental factors and lifestyle [59]. An interesting finding of the present study was that seven taxa enriched in PwMS were more abundant in females, whereas three taxa reduced in MS patients were more abundant in males. Gender differences of some of these microbiota have been reported in non-MS studies, including *Bifidobacterium* spp. [65], *Bifidobacterium* [66], and *Ruminococcus* [66,67], all with higher abundance in females. Since the female/male proportions were similar in PwMS and HCs in this study, our results contribute to the assumption that the microbiome may be involved in the well-known gender difference in MS, an idea supported by observations in animal models of autoimmunity [68]. In EAE, estrogen protects from disease onset and progression, prevents EAE-associated dysbiosis of the microbiota, and promotes abundance of microbes associated with induction of regulatory cells [69]. In a mice model of type 1 diabetes (T1D), which is not a sex-biased disease, transfer of male microbiota to female recipient mice resulted in altered metabolic profile, elevated testosterone levels, and protection from T1D [70]. How hormones, the immune system, and microbiota interact and contribute to gender differences in MS should be further investigated.

Diet has a profound effect on the structure and function of the microbiome, and persistent dietary changes can rapidly alter the microbiome composition [56,71]. The Mediterranean diet is associated with high intake of vegetables, fruits, legumes, whole grains and dairy products, moderate intake of red wine, use of unsaturated fats such as olive oil, and limited red meat consumption [72]. In our study, the vast majority of the participants scored a low or intermediate adherence to a Mediterranean diet, in line with a previous study in Israel [73], with no difference between PwMS and HCs, including within each ethnicity group. However, we found negative correlations between MDS and the abundance of two taxa enriched in PwMS, while positive correlations were found with two taxa reduced in PwMS. The Mediterranean diet impacts the gut microbiota and metabolites, for example, through increases in beneficial short-chain fatty acids (SCFAs, e.g., acetic, propionic, and butyric acids produced by fermentation of indigestible carbohydrates like dietary fibers) [72,74]. SCFAs promote anti-inflammatory responses and enhance the integrity of the intestine epithelial barrier and the blood–brain barrier [39,75,76]. Blood levels of the major SCFAs are decreased in PwMS [77], and, in a case-control study, supplement of propionic acid increased Tregs, reduced Th1 and Th17 cells, relapse rate, and brain atrophy, and stabilized disability progression [32]. Butyrate-producing bacteria were depleted in PwMS in several studies [78]. *Prevotella* among *Bacteroidetes* and *Lachnospira* among the *Firmicutes* are major contributors to fermentation of carbohydrates and increased SCFA production, especially butyrate [74,78]. Interestingly, we found reduced abundance of two members of *Lacnospira* in PwMS, namely, *Lachnospiraceae_UCG004* and *Lachnospiraceae_NK4A136_group*, as well as reduced abundance of *Prevotella* in PwMS in the Jewish cohort. A rehabilitation program for PwMS, including dietary recommendations based upon the Mediterranean diet, promoted several modifications on the microbiota—including depletion of *Collinsella*, *Eggerthella*, and *Ruminococcus*, all enriched in PwMS in the present study—and an increase in butyrate-producing microbes [36]. In an Italian study, MDS correlated with disability scores including EDSS [79], a Mediterranean diet was associated in a Turkish study with lower fatigue severity [80], and, in a US study, a Mediterranean dietary intervention reduced fatigue and EDSS [81]. These results suggest that nutritional adjustment and improved adherence to a Mediterranean diet may beneficially rehabilitate the abundance of altered microbes in PwMS and potentially reduce disease activity. We also identified correlations between specific nutrient intake and abundance of MS-differential taxa and, accordingly, outlined nutritional recommendations that may potentially promote the restoration of a “healthy” microbiome, supporting the conclusion that personalized dietary recommendations may be beneficial as an integrated part of MS therapy [71]. A clinical trial on omega-3 and omega-6 supplement on clinical outcome in PwMS showed a positive trend in favor of patients treated with omega-3 [82]. A high-vegetable/low-protein diet reduced relapse rate and EDSS and increased butyrate-producing bacterium *Lachnospiraceae*, Treg differentiation, and TGFβ and IL-10 production in PwMS [83]. Other dietary regimes with potential benefits in MS may include intermittent fasting, ketogenic diet, plant-based diet, and Mediterranean diet, as elaborated above [39]. Among the recommended nutritional adjustments, vitamin A promotes anti-inflammatory effects [39], and serum retinol inversely correlated with MRI outcomes in PwMS [84]. Increased consumption of vitamin A may also contribute to the benefits of optimizing vitamin D, which, through the complex *retinoid* X receptor-retinoic acid/vitamin D receptor, inhibits proinflammatory NF-κB [56]. The amino acid cysteine is an essential building block for sulphur-containing compounds like glutathione, an antioxidant with a major role in counteracting oxidative damage in the CNS [2] and deficient in the brain of PwMS [85]. Our data indicate benefits from increased cysteine intake, which correlated negatively with MS-enriched *Eggerthella*, a microbe associated with cysteine degradation [86]. Dietary fiber intake, as recommended, correlates negatively with the abundance of *Collinsella* [87], enriched in PwMS in our study, a microbe associated with increased proinflammatory IL-17 and intestine permeability [88,89], and dietary fibers also increase SCFA production, as detailed above [75]. Moreover, our data suggest that reducing intake of saturated trans fatty acids may increase abundance of three taxa reduced in PwMS, belonging to butyrate producers *Ruminococcaceae*, *Bacteroidetes*, and *Clostridium* [2,19]. Beneficial effects of the recommended nutritional adjustments depend upon quantity and relative ratio.

We identified KOs and KEGG pathways that differed between patients and HCs, indicating changes in the metabolic function of microbiota in PwMS. The top enriched pathway was biosynthesis of amino acids, supporting a previous report of enriched pathways involving aromatic amino acids biosynthesis in pediatric MS, associated with disease worsening [38], while another study found abnormalities in aromatic amino acid metabolites in association with disability in PwMS [90]. Possible amino acid pathways may include cysteine metabolism and accompanying glutathione level [2], as described above. Moreover, tryptophan metabolism and accompanying levels of indole-based compounds promote anti-inflammatory effects [3,91], and tryptophan supplementation reduces EAE severity [92]. The enrichment of the peptidoglycan biosynthesis pathway is interesting, since peptidoglycan, a major component of the Gram-positive bacterial cell wall with proinflammatory properties, was found in antigen-presenting cells in the brains of PwMS, and peptidoglycan-specific antibodies or plasma cells were found in the CSF and brain in PwMS [93]. Biomarker analysis (LEfSe) revealed 16 KOs, all enriched in MS, of which 7 KOs were related to ABC (ATP-binding cassette) transporters, which transport substrates across cellular membranes, and 6 KOs were related to two-component systems, regulatory mechanisms of stimuli, and response to environmental conditions. Enrichments of ABC transporter pathways in PwMS confirms two previous reports [21,23], and administration of probiotic LBS supplements was associated with reduction in KEGG ABC transporters pathways in MS [21].

The strengths of this study are the inclusion of restrictedly treatment-naïve patients with short disease duration and a cohort belonging to a small geographic area with relatively defined populations, reducing interindividual variations. The limitations of this study include the constrained sample size—like most published studies in MS, self-reported questionnaires on dietary data—which may have limited accuracy, and limited taxonomic resolution of the 16S rRNA amplicon sequencing method. Our comparative study is observational, associative, and exploratory, but findings serve as potential candidates for further research and validation as well as for functional analyses to establish causal and prognostic effects [94].

## 4. Materials and Methods

### 4.1. Recruitment of Participant and Sample Collection

A total of 57 treatment-naïve patients diagnosed with definite MS—according to the latest McDonald criteria [95]—of the relapsing–remitting subtype (RRMS) and 43 healthy controls (HC), matched for age and gender, were recruited at the MS center, Carmel Medical Center, Israel, following a protocol approved by the Institutional Review Board (0034-13-CMC, 26 December 2013), and all participants provided written informed consent. Inclusion criteria were age 18–67 years, without antibiotics/probiotics/corticosteroids treatment within the last month, and no irritable bowel disease (IBD), other autoimmune disease, or history of gastric/bowel surgery. Healthy participants had no known relative with MS up to 2nd degree. Fecal samples were obtained using a stool preservative tube (Norgen Biotek, Thorold, ON, Canada), frozen immediately at arrival at clinic, and kept at −80° until DNA extraction. All participants completed a food frequency questionnaire (FFQ) and a Mediterranean diet score (MDS) questionnaire. Demographic and clinical data were recorded. The FFQs were analyzed at the Department of Public Health, Faculty of Health Sciences, Ben-Gurion University of the Negev, Israel, for daily intake of energy and 74 various nutrients [96]. The MDS is a 17-item Mediterranean diet adherence screener, adapted to the Israeli population [97].

### 4.2. Microbial DNA Extration

Microbial DNA was extracted using the QIAamp^®^ PowerFecal^®^ Pro DNA kit (Qiagen, Tegelen, The Netherlands), according to manufacturer’s protocol. DNA 16S rRNA sequencing was performed at Hy Laboratories Ltd. (Rehovot, Israel). The V3V4 region of the 16S rRNA gene was amplified using primers from The Earth Microbiome Project. Access Array primers for Illumina (Fluidigm, San Francisco, CA, USA) were used to add adaptor and index sequences, and the reactions cleaned using Kapa Pure beads (Kapa, Roche, Basel, Switzerland). The concentration of each library was measured by Qubit (Life Technologies, Waltham, MA, USA) using the Denovix ds DNA HS assay (Deonvix, Wilmington, DE, USA) and samples pooled. The pooled library was sequenced on Illumina Miseq using a Miseq v2 Kit (Illumina, Eindhoven, The Netherlands) to generate 2 × 250 PE reads at a depth of 100,000 reads/sample. Reads were trimmed for adaptor sequences and quality; paired reads were merged and subjected to OTU de novo picking against the SILVA database at >97% sequence similarity using the CLC-bio software version 12.0.3 (Qiagen, Tegelen, The Netherlands). An amount of 12,176 OTUs were identified.

### 4.3. Statistical Analysis

Statistical analysis of microbiome data was performed with the MicrobiomeAnalyst web tool, version 1.0 (Xia Lab, McGill University, Montréal, QC, Canada), using the Marker Data Profiling Module (MDP), according to published protocols [16,98,99]. OTUs were filtered to include only features with ≥4 counts in at least 10% of samples. Testing for differences in α-diversity was performed using the Shannon alpha index (Mann–Whitney *U* test), comparing OTU richness and evenness, while β-diversity was calculated using the Bray–Curtis dissimilarity (PERMANOVA) test comparing the similarity and distance between samples on data normalized by total sum scaling (TSS). Differential analysis of individual taxa between PwMS and HCs was performed using the packages “DESeq2” (normalization by relative log expression (RLE), “EdgeR” (normalization by trimmed mean of M-values (TMM) and “MetagenomeSeq” (normalization by cumulative sum scaling (CSS). Taxa with significant differential relative abundance between PwMS and HCs in at least 2 out of 3 of the statistical methods at an adjusted *p*-value (corrected for multiple testing using the Benjamini–Hochberg false discovery rate FDR) < 0.1 were considered as differentially abundant taxa. The relative tolerant FDR was chosen given the exploratory nature of this study. Additionally, the linear discriminant analysis effective size (LEfSe) tool was used (on TSS normalized data) to determine features discriminating between PwMS and HCs, with *p*-value < 0.05, and effect size linear discriminant analysis (LDA) score > 1.8 as criteria.

Differences in gender, age, ethnicity, MDS, BMI, proportion of smokers and vegetarians, and the average intake of 75 nutrients between PwMS and HCs were assessed by the Mann–Whitney *U* test or Kruskal–Wallis test for continuous or ordinal variables and by Fischer’s exact test for nominal, categorical variables, using IBM SPSS statistics (v28), at *p*-value < 0.05.

Spearman correlations between the relative abundance of MS-differential taxa, normalized by TSS, and clinical or environmental data (dietary data (MDS or daily nutrient intake), disease severity (EDSS), or level of vitamin D) were calculated using IBM SPSS statistics (v28) at *p*-value < 0.05. Differences in relative abundance of MS-differential taxa between genders was assessed by the EdgeR and DeSeq2 packages in MicrobiomeAnalyst at FDR < 0.1.

### 4.4. Functional Analysis

The Tax4Fun2 package [17] under the Marker Data Profiling Module in the MicrobiomeAnalyst web tool was used to predict functional profiling of the corresponding microbiota, applied to filtered OTUs (1295), scaled by TSS, and transformed into 6277 Kyoto Encyclopedia of Genes and Genomes (KEGG) orthologies (KOs). The relative KO abundance table was uploaded into the Shotgun Data Profiling (SDP) module for enrichment analysis and metabolic network mapping. KOs were filtered for ≥4 in at least 10% of samples, and remaining 5472 KOs were scaled by CSS. Enrichment analysis of functional associations of KEGG pathways was calculated at FDR < 0.1. Differentially functional pathways between PwMS and HCs were identified using the Kruskal–Wallis rank test, at FDR < 0.1, and significant KOs mapped to enriched networks at FDR < 0.05. LEfSe was performed on KOs to detect metabolic functions that best distinguished between groups at FDR < 0.1.

## 5. Conclusion

We have identified alterations in the microbiome in PwMS among Israeli diverse populations, including ethnicity-specific alterations, emphasizing that an MS-associated microbiome is in part population-specific. We have revealed MS-differential microbes that correlate with gender, vitamin D levels, Mediterranean diet score, specific nutrient intake, and MS-related disability and have mapped related functional pathways. Our results support the perception that microbiome modulation, through dietary interventions, probiotics, fecal transplant, etc., could be potentially beneficial as part of integrative personalized care in MS.

## Figures and Tables

**Table 1:** Demographic and clinical data of participants.

	MS (N = 57)	Healthy Controls (N = 43)	*p*-Value
**Age**	33.6 ± 1.4	38.1 ± 1.8	0.06
**Female (%)**	70.2	58.1	0.2
**BMI (kg/m^2^)**	25.4 ± 0.6	24.7 ± 0.5	0.6
**Smoking (%)**	35.1	25.6	0.3
**Vegetarian N (%)**	1 (1.8)	1 (2.3)	0.8
**Ethnicity N (%)**			0.06
Jewish	31 (54.4)	32 (74.4)	
Arab	26 (45.6)	11 (25.6)	
**MDS**	6.95 ± 0.3	7.33 ± 0.4	0.4
MDS N (%)			0.6
Low (≤6)	27 (47.4)	18 (41.9)	
Intermediate (7–11)	27 (47.4)	22 (51.2)	
High (≥12)	3 (5.3)	3 (7.0)	
**Vitamin D (ng/mL)**	44.5 ± 3.7		
**Disease Duration (months)**	2.6 ± 0.6		
**EDSS mean ± SE**	1.56 ± 0.14		
**Annual relapse rate mean ± SE**	0.85 ± 0.07		

Abbreviation: BMI—body mass index, EDSS—expanded disability status scale, MDS—Mediterranean diet score, SE—standard error.

**Table 2:** Differential abundant taxa between PwMS and HCs.

Differentially Abundant Taxa	EdgeR		DeSeq2		Metag.Seq	LefSe		Highest
	FDR	FC	FDR	FC	FDR	*p*-Value	LDA	in
**Order**								
*Aeromonadales*	0.059	4.7	0.002	26.1	ns	ns	-	MS
*Enterobacteriales*	ns	-	ns	-	ns	0.007	−4.1	HC
**Family**								
*Clostridium_sp__K4410_MGS_306*	0.019	0.31	ns	-	0.022	ns	-	HC
*Enterobacteriaceae*	ns	-	ns	-	ns	0.007	−4.1	HC
*gut_metagenome*	1.10^−7^	0.18	ns	-	1.10^−5^	0.018	−3.4	HC
*Peptostreptococcaceae*	ns	-	ns	-	ns	0.048 *	4.3 *	MS
*Succinivibrionaceae*	0.055	4.5	0.002	42.8	ns	ns	-	MS
**Genus**								
*Azospirillum_sp__47_25*	3.39^−7^	0.16	ns	-	0.021	ns	-	HC
*Barnesiella*	ns	-	ns	-	ns	0.034	3.1	MS
*Collinsella*	ns	-	ns	-	0.096	0.045 *	4.5 *	MS
*Eggerthella*	ns	-	ns	-	ns	0.014	1.8	MS
*Enterobacter*	0.013	3.5	ns	-	ns	0.020	3.4	MS
*Escherichia_Shigella*	0.003 ^Ϯ^	0.18 ^Ϯ^	ns	-	ns	0.018	−4.1	HC
*Flavonifractor*	0.0006	4.0	0.077	2.5	ns	ns	-	MS
*gut_metagenome*	1.59^−6^	0.20	ns	-	6.28^−5^	0.018	−3.4	HC
*Lachnospiraceae_UCG_003*	0.0007	3.6	ns	-	0.023	ns	ns	MS
*Lachnospiraceae_UCG_004*	ns	-	ns	-	ns	0.007	−4.0	HC
*Lachnospiraceae_NK4A136_group*	0.016	0.53	0.085 *	0.58 *	ns	ns	-	HC
*Merdibacter*	0.0014	2.5	0.036	3.7	0.0005	ns	-	MS
*Mitsuokella*	6.88^−7^	13.6	5.02^−6^	56.3	1.25^−5^	0.048	4.1	MS
*Negativibacillus*	0.013	2.2	0.099	2.3	0.049	0.042	3.0	MS
*Olsenella*	0.050	2.5	ns	-	0.023	0.031	3.6	MS
*Peptococcus*	0.054	0.47	0.032 *	0.22 *	ns	ns	-	HC
*Romboutsia*	ns	-	ns	-	ns	0.017	4.3	MS
*Ruminiclostridium_5*	ns	-	ns	-	ns	0.049	−3.8	HC
*Ruminococcaceae_UCG_013*	0.055	0.60	0.099	0.60	ns	0.011	−3.9	HC
*Ruminococcus_gnavus_group*	ns	-	4.77^−7^	7.5	ns	0.019	3.5	MS
*Sellimonas*	0.006	3.3	ns	-	0.050	ns	-	MS
*Succinivibrio*	0.008	6.8	0.009	57.0	0.060	ns	-	MS
*Tyzzerella_4*	1.59^−7^	0.12	ns	-	0.088	ns	-	HC
**Species**								
*Bacteroides_timonensis*	ns	-	ns	-	ns	0.019	−2.4	HC
*Bifidobacterium_animalis*	0.011	0.44	ns	-	0.002	ns	-	HC
*Bifidobacterium_sp__MC_10*	0.092	1.8	ns	-	0.003	ns	-	MS
*Clostridium_sp__K4410_MGS_306*	0.001	0.24	ns	-	0.037	ns	-	HC
*Escherichia_coli*	ns	-	0.043	2.9	ns	0.024	−3.9	HC
*Negativibacillus_massiliensis*	0.0003	3.6	ns	-	8.01^−5^	ns	-	MS
*Ruminococcus_gnavus_CC55_001C*	0.099	2.6	0.0003	18.4	0.071	0.009	3.5	MS
*Streptococcus_thermophilus_TH1435*	ns	-	ns	-	ns	0.028	−3.6	HC
*uncultured_Bacteroidetes_bacterium*	0.001	2.5	0.032	3.6	0.016	ns	-	MS
*uncultured_Clostridium_sp_1*	0.085	0.6	0.053 ^Ϯ^	0.6	ns	0.013	−3.9	HC

Taxa with differential relative abundance in at least two out of three tests (DESeq2, MetagenomeSeq, and EdgeR) at FDR < 0.1 or identified as taxa discriminating between PwMS and HCs by LEfSe at *p*-value < 0.5 and LDA > 1.8. * Identified after filtration of ≥4 reads in ≥20% of samples and ≥10% variance. ^Ϯ^ Identified after filtration of ≥5 reads in ≥50% of samples and ≥20% variance. Abbreviations: FC—fold change, FDR—false discovery rate, HCs—healthy controls, LDA—linear discriminant analysis, ns—not significant.

## Data Availability

All relevant data are available within the article or supplementary material or will be made available from the corresponding author upon request from any qualified investigator.

## Supplementary Materials

The following supporting information can be downloaded at: https://www.mdpi.com/article/10.3390/ijms241915024/s1.

## References

[1] Olsson T., Barcellos L.F., Alfredsson L. (2016). Interactions between genetic, lifestyle and environmental risk factors for multiple sclerosis. Nat. Rev. Neurol..

[2] Dopkins N., Nagarkatti P.S., Nagarkatti M. (2018). The role of gut microbiome and associated metabolome in the regulation of neuroinflammation in multiple sclerosis and its implications in attenuating chronic inflammation in other inflammatory and autoimmune disorders. Immunology.

[3] Cox L.M., Weiner H.L. (2018). Microbiota Signaling Pathways that Influence Neurologic Disease. Neurotherapeutics.

[4] Cox L.M., Abou-el-hassan H., Maghzi A.H., Vincentini J., Weiner H.L. (2019). The sex-specific interaction of the microbiome in neurodegenerative diseases. Brain Res..

[5] Rojas M., Restrepo-Jiménez P., Monsalve D.M., Pachecoa Y., Acosta-Ampudiaa Y., Ramírez-Santanaa C., Leungc P.S.C., Ansaric A.A., Gershwinc M.E., Anaya J. (2018). Molecular mimicry and autoimmunity. J. Autoimmun..

[6] Mirza A., Forbes J.D., Zhu F., Bernstein C.N., Domselaar G.V., Graham M., Waubante E., Tremlett H. (2020). The multiple sclerosis gut microbiota: A systematic review. Mult. Scler. Relat. Disord..

[7] Berer K., Mues M., Koutrolos M., AlRasbi Z., Boziki M., Johner C., Wekerle H., Krishnamoorthy G., Rasbi Z.A., Boziki M. (2011). Commensal microbiota and myelin autoantigen cooperate to trigger autoimmune demyelination—With comments. Nature.

[8] Cekanaviciute E., Yoo B.B., Runia T.F., Debelius J.W., Singh S., Nelson C.A., Kanner R., Bencosme Y., Lee Y.K., Hauser S.L. (2017). Gut bacteria from multiple sclerosis patients modulate human T cells and exacerbate symptoms in mouse models. Proc. Natl. Acad. Sci. USA.

[9] Berer K., Gerdes L.A., Cekanaviciute E., Jiac X., Xiaod L., Xiad Z., Liud C., Klotze L., Staufferf U., Baranzini S.E. (2017). Gut microbiota from multiple sclerosis patients enables spontaneous autoimmune encephalomyelitis in mice. Proc. Natl. Acad. Sci. USA.

[10] Cresci G.A., Bawden E. (2015). Gut microbiome: What we do and don’t know. Nutr. Clin. Pract..

[11] Pinart M., Dötsch A., Schlicht K., Laudes M., Bouwman J., Forslund S.K., Pischon T., Nimptsch K. (2022). Gut microbiome composition in obese and non-obese persons: A systematic review and meta-analysis. Nutrients.

[12] Cantoni C., Lin Q., Dorsett Y., Ghezzi L., Liu Z., Pan Y., Chen K., Han Y., Li Z., Xiao H. (2022). Alterations of host-gut microbiome interactions in multiple sclerosis. EBioMedicine.

[13] Nearing J.T., Douglas G.M., Hayes M.G., MacDonald J., Desai D.K., Allward N., Jones C.M.A., Wright R.J., Dhanani A.S., Comeau A.M. (2022). Microbiome differential abundance methods produce different results across 38 datasets. Nat. Commun..

[14] World Medical Association https://www.wma.net/policies-post/wma-statement-on-vitamin-d-insufficiency/.

[15] Filippi M., Bar-Or A., Piehl F., Preziosa P., Solari A., Vukusic S., Rocca M.A. (2018). Multiple Sclerosis. Nat. Rev. Dis. Prim..

[16] Chong J., Liu P., Zhou G., Xia J. (2020). Using MicrobiomeAnalyst for comprehensive statistical, functional, and meta-analysis of microbiome data. Nat. Protoc..

[17] Wemheuer F., Taylor J.A., Daniel R., Johnston E., Meinicke P., Thomas T., Wemheuer B. (2020). Tax4Fun2: Prediction of habitat-specific functional profiles and functional redundancy based on 16S rRNA gene sequences. Environ. Microbiomes.

[18] Tremlett H., Fadrosh D.W., Faruqi A.A., Zhu F., Hart J., Roalstad S., Graves J., Lynch S., Waubant E., on behalf of the US Network of Pediatric MS Centers (2016). Gut microbiota in early pediatric multiple sclerosis: A case-control study. Eur. J. Neurol..

[19] Tremlett H., Zhu F., Arnold D., Bar-Or A., Bernstein C.N., Bonner C., Forbes J.D., Graham M., Hart J., Knox N.C. (2021). The gut microbiota in pediatric multiple sclerosis and demyelinating syndromes. Ann. Clin. Transl. Neurol..

[20] Yadav M., Ali S., Shrode R.L., Shahi S.K., Jensen S.N., Hoang J., Cassidy S., Olalde H., Guseva N., Paullus M. (2022). Multiple sclerosis patients have an altered gut mycobiome and increased fungal to bacterial richness. PLoS ONE.

[21] Tankou S.K., Regev K., Healy B.C., Tjon E., Laghi L., Cox L.M., Kivisäkk P., Pierre I.V., Hrishikesh L., Gandhi R. (2018). A probiotic modulates the microbiome and immunity in multiple sclerosis. Ann. Neurol..

[22] Jangi S., Gandhi R., Cox L.M., Li N., von Glehn F., Yan R., Patel B., Mazzola M.A., Liu S., Glanz B.L. (2016). Alterations of the human gut microbiome in multiple sclerosis. Nat. Commun..

[23] Chen J., Chia N., Kalari K.R., Yao J.Z., Novotna M., Soldan M.M.P., Luckey D.H., Marietta E.V., Jeraldo P.R., Chen X. (2016). Multiple sclerosis patients have a distinct gut microbiota compared to healthy controls. Sci. Rep..

[24] Miyake S., Kim S.S.W.S., Suda W., Oshima K., Nakamura M., Matsuoka T., Chihara N., Tomita A., Sato W., Kim S.W. (2015). Dysbiosis in the gut microbiota of patients with multiple sclerosis, with a striking depletion of species belonging to clostridia XIVa and IV clusters. PLoS ONE.

[25] Ventura R.E., Iizumi T., Battaglia T., Liu M., Perez-Perez G.I., Herbert I., Blaser M.J. (2019). Gut microbiome of treatment-naïve MS patients of different ethnicities early in disease course. Sci. Rep..

[26] Central Bureau of Statistics Population of Israel on the Eve of 2023. https://www.cbs.gov.il/en/mediarelease/pages/2022/population-of-israel-on-the-eve-of-2023.aspx.

[27] Alter M., Kahana E., Zilber N., Ariel Miller and for the Israeli MS Study Group (2006). Multiple sclerosis frequency in Israel’s diverse populations. Neurology.

[28] Benedek G., Paperna T., Avidan N., Lejbkowicz I., Oksenberg J.R., Wang J., Brautbar C., Israel S., Miller A. (2010). Opposing effects of the HLA-DRB1*0301-DQB1*0201 haplotype on the risk for multiple sclerosis in diverse Arab populations in Israel. Genes. Immun..

[29] Siegel M., Paperna T., Lejbkowicz I., Petrou P., Shahien R., Karussis D., Lavi I., Dishon S., Rawashdeh H. (2012). Miller, AMultiple sclerosis in diverse populations: Characteristics in distinct Arab ethnicities in Israel. Mult. Scler..

[30] Budhram A., Parvathy S., Kremenchutzky M., Silverman M. (2017). Breaking down the gut microbiome composition in multiple sclerosis. Mult. Scler. J..

[31] Ling Z., Cheng Y., Yan X., Shao L., Liu X., Zhou D., Zhang L., Yu K., Zhao L. (2020). Alterations of the Fecal Microbiota in Chinese Patients With Multiple Sclerosis. Front. Immunol..

[32] Duscha A., Gisevius B., Hirschberg S., Yissachar N., Stangl G.I., Eilers E., Bader V., Haase S., Kaisler J., David C. (2020). Propionic Acid Shapes the Multiple Sclerosis Disease Course by an Immunomodulatory Mechanism. Cell.

[33] Reynders T., Devolder L., Valles-Colomer M., Van Remoortel A., Joossens M., De Keyser J., Nagels G., D’hooghe M., Raes J. (2020). Gut microbiome variation is associated to Multiple Sclerosis phenotypic subtypes. Ann. Clin. Transl. Neurol..

[34] Cox L.M., Maghzi A.H., Liu S., Tankou S.K., Dhang F.H., Willocq V., Song A., Wasén C., Tauhid S., Chu R. (2021). Gut Microbiome in Progressive Multiple Sclerosis. Ann. Neurol..

[35] Cao R.R., He P., Lei S.F. (2021). Novel microbiota-related gene set enrichment analysis identified osteoporosis associated gut microbiota from autoimmune diseases. J. Bone Miner. Metab..

[36] Barone M., Mendozzi L., Amico F.D., Saresella M., Rampelli S., Piancone F., La Rosa F., Marventano I., Clerici M., d’Arma A. (2021). Influence of a High-Impact Multidimensional Rehabilitation Program on the Gut Microbiota of Patients with Multiple Sclerosis. Int. J. Mol. Sci..

[37] Ezendam J., De Klerk A., Gremmer E.R., van Loveren H. (2008). Effects of Bifidobacterium animalis administered during lactation on allergic and autoimmune responses in rodents. Clin. Exp. Immunol..

[38] Horton M.K., Mccauley K., Fadrosh D., Fujimura K., Graves J., Ness J., Wheeler Y., Gorman M.P., Benson L.A., Weinstock-Guttman B. (2021). Gut microbiome is associated with multiple sclerosis activity in children. Ann. Clin. Transl. Neurol..

[39] Sanchez J.M.S., DePaula-Silva A.B., Libbey J.E., Fujinami R.S. (2022). Role of diet in regulating the gut microbiota and multiple sclerosis. Clin. Immunol..

[40] Gomaa E.Z. (2020). Human gut microbiota/microbiome in health and diseases: A review. Antonie Van Leeuwenhoek. Int. J. General. Mol. Microbiol..

[41] Katz Sand I., Zhu Y., Ntranos A., Clemente J.C., Cekanaviciute E., Brandstadter R., Crabtree-Hartman E., Singh S., Bencosme Y., Debelius J. (2019). Disease-modifying therapies alter gut microbial composition in MS. Neurol. Neuroimmunol. NeuroInflamm..

[42] Levi I., Gurevich M., Perlman G., Magalashvili D., Menascu S., Bar B., Godneva A., Zahavi L., Chermon D., Kosower N. (2021). Potential role of indolelactate and butyrate in multiple sclerosis revealed by integrated microbiome-metabolome analysis. Cell Reports Med..

[43] Johnson J.S., Spakowicz D.J., Hong B.Y., Petersen L.M., Demkowicz P., Chen L., Leopold S.R., Hanson B.M., Agresta H.O., Gerstein M. (2019). Evaluation of 16S rRNA gene sequencing for species and strain-level microbiome analysis. Nat. Commun..

[44] Dash S., Syed Y.A., Khan M.R. (2022). Understanding the Role of the Gut Microbiome in Brain Development and Its Association With Neurodevelopmental Psychiatric Disorders. Front. Cell Dev. Biol..

[45] Pinna N.K., Anjana R.M., Saxena S., Dutta A., Gnanaprakash V., Rameshkumar G., Aswath S., Raghavan S., Rani C.S.S., Radha V. (2021). Trans-ethnic gut microbial signatures of prediabetic subjects from India and Denmark. Genome Med..

[46] Tecer D., Gogus F., Id A.K., Erdogan M., Hasanreisoglu M., Ergin A., Karakan T., Kozan R., Coban S., Diker K.S. (2020). Succinivibrionaceae is dominant family in fecal microbiota of Behcet’s Syndrome patients with uveitis. PLoS ONE.

[47] Cosorich I., Dalla-Costa G., Sorini C., Ferrarese R., Messina M.J., Dolpady J., Radice E., Mariani A., Testoni P.A., Canducci F. (2017). High frequency of intestinal T H 17 cells correlates with microbiota alterations and disease activity in multiple sclerosis. Sci. Adv..

[48] Karni A., Kahana E., Zilber N., Abramsky O., Alter M., Karussis D. (2003). The frequency of multiple sclerosis in Jewish and Arab populations in greater Jerusalem. Neuroepidemiology.

[49] Saaroni H., Sigal A., Lejbkowicz I., Miller A. (2010). Mediterranean weather conditions and exacerbations of multiple sclerosis. Neuroepidemiology.

[50] Tremlett H., Fadrosh D.W., Faruqi A.A., Hart J., Roalstad S., Graves J., Lynch S., Waubant E., Aaen G., Belman A. (2016). Gut microbiota composition and relapse risk in pediatric MS: A pilot study. J. Neurol. Sci..

[51] Sintzel M.B., Rametta M., Reder A.T. (2018). Vitamin D and Multiple Sclerosis: A Comprehensive Review. Neurol. Ther..

[52] Pierrot-deseilligny C., Souberbielle J. (2010). Is hypovitaminosis D one of the environmental risk factors for multiple sclerosis?. Brain.

[53] Golan D., Staun-Ram E., Glass-Marmor L., Lavi I., Rozenberg O., Dishon S., Barak M., Ish-Shalom S., Miller A. (2013). The influence of vitamin D supplementation on melatonin status in patients with multiple sclerosis. Brain Behav. Immun..

[54] Golan D., Halhal B., Glass-Marmor L., Staun-Ram E., Rozenberg O., Lavi I., Dishon S., Barak M., Ish-Shalom S., Miller A. (2013). Vitamin D supplementation for patients with multiple sclerosis treated with interferon-beta: A randomized controlled trial assessing the effect on flu-like symptoms and immunomodulatory properties. BMC Neurol..

[55] Ghareghani M., Reiter R.J., Zibara K., Farhadi N. (2018). Latitude, Vitamin D, Melatonin, and Gut Microbiota Act in Concert to Initiate Multiple Sclerosis: A New Mechanistic Pathway. Front. Immunol..

[56] Riccio P., Rossano R. (2018). Diet, Gut Microbiota, and Vitamins D + A in Multiple Sclerosis. Neurotherapeutics.

[57] Petrak F., Herpertz S., Hirsch J., Röhrig B., Donati-Hirsch I., Juckel G., Meier J.J., Gatermann S. (2022). Gut microbiota differs in composition between adults with type 1 diabetes with or without depression and healthy control participants: A case-control study. BMC Microbiol..

[58] Alvarez-Sanchez N., Dunn S.E. (2023). Potential biological contributers to the sex difference in multiple sclerosis progression. Front. Immunol..

[59] Harbo H.F., Gold R., Tintora M. (2013). Sex and gender issues in multiple sclerosis. Ther. Adv. Neurol. Disord..

[60] Fish E. (2008). The X-files in immunity: Sex-based differences predispose immune responses. Nat. Rev. Immunol..

[61] Rizzetto L., Fava F., Tuohy K.M., Selmi C. (2018). Connecting the immune system, systemic chronic inflammation and the gut microbiome: The role of sex. J. Autoimmun..

[62] Gold S.M., Voskuhl R.R. (2009). Estrogen and testosterone therapies in multiple sclerosis. Prog. Brain Res..

[63] Bouman A., Heineman M.J., Faas M.M. (2005). Sex hormones and the immune response in humans. Hum. Reprod. Update.

[64] Selmi C. (2008). The X in sex: How autoimmune diseases revolve around sex chromosomes. Best. Pract. Res. Clin. Rheumatol..

[65] Borgo F., Garbossa S., Riva A., Severgnini M., Luigiano C., Benetti A., Pontiroli A.E., Morace G., Borghi E. (2018). Body Mass Index and Sex Affect Diverse Microbial Niches within the Gut. Front. Microbiol..

[66] Takagi T., Naito Y., Inoue R., Kashiwagi S., Uchiyama K., Mizushima K., Tsuchiya S., Dohi O., Yoshida N., Kamada K. (2019). Differences in gut microbiota associated with age, sex, and stool consistency in healthy Japanese subjects. J. Gastroenterol..

[67] Gao X., Zhang M., Xue J., Huang J., Zhuang R., Zhou X., Zhang H., Fu Q., Hao Y. (2018). Body Mass Index Differences in the Gut Microbiota Are Gender Specific. Front. Microbiol..

[68] Ortana E., Pierdominici M., Maselli A., Veroni C., Aloisi F., Shoenfeld Y. (2016). Sex-based differences in autoimmune diseases. Ann. Ist. Super. Sanità.

[69] Benedek G., Zhang J., Nguyen H., Kent G., Seifert H.A., Davin S., Stauffer P., Vandenbark A.A., Karstens L., Asquith M. (2017). Estrogen protection against EAE modulates the microbiota and mucosal-associated regulatory cells. J. Neuroimmunol..

[70] Markle J.G.M., Frank D.N., Mortin-Toth S., Robertson C.E., Feazel L.M., Rolle-Kampczyk U., Von Bergen M., McCoy K.D., Macpherson A.J., Danska J.S. (2013). Sex differences in the gut microbiome drive hormone-dependent regulation of autoimmunity. Science (80-).

[71] Stoiloudis P., Kesidou E., Bakirtzis C., Sintila S.A., Konstantinidou N., Boziki M., Grigoriadis N. (2022). The Role of Diet and Interventions on Multiple Sclerosis: A Review. Nutrients.

[72] Gutiérrez-Díaz I., Fernández-Navarro T., Sánchez B., Margolles A., González S. (2016). Mediterranean diet and faecal microbiota: A transversal study. Food Funct..

[73] Bilenko N., Fraser D., Vardi H., Shai I., Shahar D.R. (2005). Mediterranean diet and cardiovascular diseases in an Israeli population. Prev. Med..

[74] De Filippis F., Pellegrini N., Vannini L., Jeffery I.B., La Storia A., Laghi L., Serrazanetti D.I., Di Cagno R., Ferrocino I., Lazzi C. (2015). High-level adherence to a Mediterranean diet beneficially impacts the gut microbiota and associated metabolome. Gut.

[75] Haghikia A., Jörg S., Duscha A., Berg J., Manzel A., Waschbisch A., Hammer A., Lee D.H., May C., Wilck N. (2015). Dietary Fatty Acids Directly Impact Central Nervous System Autoimmunity via the Small Intestine. Immunity.

[76] Elsayed N.S., Aston P., Bayanagari V.R., Shukla S.K. (2022). The gut microbiome molecular mimicry piece in the multiple sclerosis puzzle. Front. Immunol..

[77] Park J., Wang Q., Wu Q., Mao-Draayer Y., Kim C.H. (2019). Bidirectional regulatory potentials of short-chain fatty acids and their G-protein-coupled receptors in autoimmune neuroinflammation. Sci. Rep..

[78] Noto D., Miyake S. (2022). Gut dysbiosis and multiple sclerosis. Clin. Immunol..

[79] Esposito S., Sparaco M., Maniscalco G.T., Signoriello E., Signoriello E., Lanzillo R., Russo C., Carmisciano L., Cepparulo S., Lavorgna L. (2021). Lifestyle and Mediterranean diet adherence in a cohort of Southern Italian patients with Multiple Sclerosis. Mult. Scler. Relat. Disord..

[80] Öztürk Y.E., Helvaci E.M., Kaya P.S., Terzi M. (2023). Is Mediterranean diet associated with multiple sclerosis related symptoms and fatigue severity?. Nutr. Neurosci..

[81] Katz Sand I., Benn E.K.T., Fabian M., Fitzgerald K.C., Digga E., Deshpande R., Miller A., Gallo S., Arab L. (2019). Randomized-controlled trial of a modified Mediterranean dietary program for multiple sclerosis: A pilot study. Mult. Scler. Relat. Disord..

[82] Bates D.E., Cartlidge N.E.F., French J.M., Jackson M.J., Nightingale S., Shaw D.A., Smith S., Woo E., Hawkins S.A., Millar J.H.D. (1989). A double-blind controlled trial of long chain n-3 polyunsaturated fatty acids in the treatment of multiple sclerosis. J. Neurol. Neurosurg. Psychiatry.

[83] Saresella M., Mendozzi L., Rossi V., Mazzali F., Piancone F., LaRosa F., Marventano I., Caputo D., Felis G.E., Clerici M. (2017). Immunological and clinical effect of diet modulation of the gut microbiome in multiple sclerosis patients: A pilot study. Front. Immunol..

[84] Løken-Amsrud K.I., Myhr K.M., Bakke S.J., Beiske A.G., Bjerve K.S., Bjørnarå B.T., Hovdal H., Lilleås F., Midgard R., Pedersen T. (2013). Retinol levels are associated with magnetic resonance imaging outcomes in multiple sclerosis. Mult. Scler. J..

[85] Cho G., Ritzmann F., Eckstein M., Huch M., Briviba K., Behsnilian D., Neve H., Franz C.M.A.P. (2016). Quantification of Slackia and Eggerthella spp. in Human Feces and Adhesion of Representatives Strains to Caco-2 Cells. Front. Microbiol..

[86] Braccia D.J., Jiang X., Pop M., Hall A.B. (2021). The Capacity to Produce Hydrogen Sulfide (H_2_S) via Cysteine Degradation Is Ubiquitous in the Human Gut Microbiome. Front. Microbiol..

[87] Gomez-Arango L.F., Barrett H.L., Wilkinson S.A., Callaway L.K., McIntyre H.D., Morrison M., Dekker Nitert M. (2018). Low dietary fiber intake increases Collinsella abundance in the gut microbiota of overweight and obese pregnant women. Gut Microbes.

[88] Mena-Vázquez N., Ruiz-Limón P., Moreno-Indias I., Manrique-Arija S., Tinahones F.J., Fernández-Nebro A. (2020). Expansion of rare and harmful lineages is associated with established rheumatoid arthritis. J. Clin. Med..

[89] Chen J., Wright K., Davis J.M., Jeraldo P., Marietta E.V., Murray J., Nelson H., Matteson E.L., Taneja V. (2016). An expansion of rare lineage intestinal microbes characterizes rheumatoid arthritis. Genome Med..

[90] Fitzgerald K.C., Smith M.D., Kim S., Sotirchos E.S., Kornberg M.D., Douglas M., Nourbakhsh B., Graves J., Rattan R., Poisson L. (2021). Multi-omic evaluation of metabolic alterations in multiple sclerosis identifies shifts in aromatic amino acid metabolism. Cell Rep. Med..

[91] Quintana F.J., Basso A.S., Iglesias A.H., Korn T., Farez M.F., Bettelli E., Caccamo M., Oukka M., Weiner H.L. (2008). Control of T reg and T H 17 cell differentiation by the aryl hydrocarbon receptor. Nature.

[92] Rothhammer V., Mascanfroni I.D., Lukas B., Takenaka M.C., Kenison J.E., Mayo L., Chao C.C., Patel B., Yan R., Blain M. (2016). Type I interferons and microbial metabolites of tryptophan modulate astrocyte activity and central nervous system inflammation via the aryl hydrocarbon receptor. Nat. Med..

[93] Schrijver I.A., Van Meurs M., Melief M.J., Ang C.W., Buljevac D., Ravid R., Hazenberg M.P., Laman J.D. (2001). Bacterial peptidoglycan and immune reactivity in the central nervous system in multiple sclerosis. Brain.

[94] Kodikara S., Ellul S., Le Cao K.A. (2022). Statistical challenges in longitudinal microbiome data analysis. Brief. Bioinform..

[95] Thompson A.J., Banwell B.L., Barkhof F., Carroll W.M., Coetzee T., Comi G., Correale J., Fazekas F., Filippi M., Freedman M.S. (2018). Diagnosis of multiple sclerosis: 2017 revisions of the McDonald criteria. Lancet Neurol..

[96] Ben-Avraham S., Kohn E., Tepper S., Lubetzky R., Mandel D., Berkovitch M., Shahar D.R. (2023). Ultra-processed food (UPF) intake in pregnancy and maternal and neonatal outcomes. Eur. J. Nutr..

[97] Abu-Saad K., Endevelt R., Goldsmith R., Freedman L.S., Ziv A., Alpert G., Atamna A., Kalter-Leibovici O. (2019). Adaptation and predictive utility of a Mediterranean diet screener score. Clin. Nutr..

[98] Dhariwal A., Chong J., Habib S., King I.L., Agellon L.B., Xia J. (2017). MicrobiomeAnalyst: A web-based tool for comprehensive statistical, visual and meta-analysis of microbiome data. Nucleic Acids Res..

[99] Lu Y., Zhou G., Ewald J., Pang Z., Shiri T., Xia J. (2023). MicrobiomeAnalyst 2.0: Comprehensive statistical, functional and integrative analysis of microbiome data. Nucleic Acids Res..

