# Supplementary material for: Multiple Sclerosis-Associated Gut Microbiome in the Israeli Diverse Populations: Associations with Ethnicity, Gender, Disability Status, Vitamin D Levels, and Mediterranean Diet
Source: Int J Mol Sci. 2023 Oct 9;24(19):15024. doi: 10.3390/ijms241915024 (PMC10573818; doi:10.3390/ijms241915024)
Supplement: Supplementary file 1 [file ijms-24-15024-s001.zip › Supplementary Material legend.pdf]

Supplementary Material legend:

Figure S1 General microbiome composition according to clinical and demographic factors. Graphs presenting **A-** alpha diversity and **B**- beta-diversity of the microbiome composition (OTU level) of all samples according to: gender, age (divided into 3 age-groups: 18-30 years, 31-43 years, 44-67 years), BMI (divided into 4 groups: underweight (<18.5), healthy weight (18.5-24.9), overweight (25-29.9) and obesity (>30)), Ethnicity (Jewish and Arabs), smoking (yes/no) and adherence to Mediterranean diet score (MDS low (1-6 points), intermediate (7-11 points), high (2-17 points).
