# Supplementary material for: Multiple Sclerosis-Associated Gut Microbiome in the Israeli Diverse Populations: Associations with Ethnicity, Gender, Disability Status, Vitamin D Levels, and Mediterranean Diet
Source: Int J Mol Sci. 2023 Oct 9;24(19):15024. doi: 10.3390/ijms241915024 (PMC10573818; doi:10.3390/ijms241915024)
Supplement: Supplementary file 1 [file ijms-24-15024-s001.zip › Table S1.pdf]

**Table S1A Taxa with differential relative abundance between PwMS and HC in the Jewish cohort**

| Differentially abundant taxa | Differential abundance methods | | | | | | | |
|---|---|---|---|---|---|---|---|---|
| | EdgeR | | DeSeq2 | | Metag.Seq | LefSe | | Highest in |
| | FDR | FC | FDR | FC | FDR | P value | LDA | |
| **Order** | | | | | | | | |
| o__Enterobacteriales | ns | - | ns | - | ns | 0.006 | 4.15 | MS |
| Family | | | | | | | | |
| f__Bacteroidaceae | ns | - | ns | - | ns | 0.038 | 5.45 | MS |
| f__Enterobacteriaceae | ns | - | ns | - | ns | 0.006 | 4.15 | MS |
| f__gut_metagenome | $3.87^{-5}$ | 0.15 | ns | - | 0.0006 | 0.031 | -3.45 | HC |
| f__Prevotellaceae | ns | - | ns | - | ns | 0.024 | -5.46 | HC |
| **Genus** | | | | | | | | |
| g__Bacteroides | ns | - | ns | - | ns | 0.038 | 5.45 | MS |
| g__Bilophila | 0.064 | 1.87 | 0.064 | 1.79 | ns | 0.01 | 3.56 | MS |
| g__Butyricimonas | ns | - | ns | - | ns | 0.020 | 3.63 | MS |
| g__DTU089 | ns | - | ns | - | ns | 0.045 | 2.03 | HC |
| g__Eggerthella | ns | - | 0.020 | 6.33 | ns | 0.011 | 2.49 | MS |
| g__Enterobacter | $1.35^{-8}$ | 24.9 | 0.035 | 13.9 | 0.043 | 0.01 | 3.8 | |
| g__Erysipelotrichaceae_UCG_003 | ns | - | ns | - | ns | 0.041 | -4.48 | HC |
| g__Escherichia_Shigella | ns | - | 0.039 | 4.07 | ns | 0.016 | 4.02 | MS |
| g__Eubacterium_xylanophilum_group | | | | | | 0.043 | -3.52 | HC |
| g__Flavonifractor | 0.0002 | 6.3979 | 0.0003 | 7.03 | ns | 0.013 | 3.75 | MS |
| g__Fusicatenibacter | ns | - | ns | - | ns | 0.021 | -4.28 | HC |
| g__gut_metagenome | 0.0002 | 0.1662 | 0.0003 | 0.02 | 0.003 | 0.031 | -3.46 | HC |
| g__Klebsiella | ns | - | 0.020 | 26.9 | 0.012 | 0.025 | -2.16 | MS |
| g__Lachnospiraceae_ND3007_group | ns | - | ns | - | ns | 0.006 | -3.9 | HC |
| g__Lachnospiraceae_UCG_001 | ns | - | ns | - | ns | 0.045 | -3.62 | HC |
| g__Lachnospiraceae_UCG_004 | ns | - | 0.04 | 0.53 | ns | 0.013 | -4.26 | HC |
| g__Lachnospiraceae_NK4A136_group | ns | - | ns | - | ns | 0.023 | -3.89 | HC |
| g__Merdibacter | 0.007 | 2.49 | 0.0631 | 3.34 | 0.043 | ns | - | MS |
| g__Mitsuokella | 0.0002 | 14.6 | $8.64^{-5}$ | 193.4 | 0.01 | ns | - | MS |
| g__Negativibacillus | 0.002 | 3.19 | 0.0317 | 3.07 | 0.043 | 0.026 | 3.2 | MS |
| g__Olsenella | ns | - | ns | - | 0.066 | 0.038 | 3.62 | MS |
| g__Oscillibacter | 0.087 | 1.92 | 0.033 | 1.98 | ns | | | MS |
| g__Prevotella_9 | ns | - | ns | - | ns | 0.034 | -5.4 | HC |
| g__Ruminococcaceae_UCG_013 | 0.083 | 0.53 | 0.04 | 0.53 | ns | 0.01 | -4.01 | HC |
| g__Ruminococcus_1 | ns | - | ns | - | ns | 0.043 | -4.14 | HC |
| **Species** | | | | | | | | |
| s__Bacteroides_ovatus_V975 | ns | - | ns | - | ns | 0.020 | -3.64 | HC |

| | | | | | | | | |
|---|---|---|---|---|---|---|---|---|
| s__Bacteroides_timonensis | ns | - | ns | - | ns | 0.04 | -2.58 | HC |
| s__Escherichia_coli | ns | - | 0.067 | 4.01 | ns | 0.026 | 4 | MS |
| s__Fusicatenibacter_saccharivorans | ns | - | ns | - | ns | 0.023 | -4.22 | HC |
| s__gut_metagenome (feature) | 0.0003 | 12.3 | ns | - | ns | 0.033 | 4.33 | MS |
| s__Negativibacillus_massiliensis | 0.0005 | 4.59 | ns | - | 0.003 | ns | - | MS |
| s__Streptococcus_salivarius_subsp__ thermophilus | ns | - | ns | - | ns | 0.038 | -3.05 | HC |
| s__Streptococcus_thermophilus_TH1 435 | ns | - | ns | - | ns | 0.04 | -3.59 | HC |
| s__uncultured_Bacteroidetes_bacteri um | 0.0001 | 3.75 | 0.01 | 6.19 | 0.001 | 0.045 | 2.81 | MS |
| s__uncultured_Clostridiaceae_bacter ium | ns | - | ns | - | ns | 0.036 | -2.49 | HC |
| s__uncultured_Clostridium_sp_ | 0.098 | 0.56 | 0.0671 | 0.55 | ns | 0.001 | -4.05 | HC |
| s__uncultured_Roseburia_sp_ | 0.083 | 0.35 | ns | - | ns | 0.042 | -3.58 | HC |
| s__uncultured_Ruminococcus_sp_ | 0.045 | 3.6 | ns | - | $2.56^{-6}$ | ns | - | MS |

Taxa with differential relative abundance in at least two out of three tests (DESeq2, MetagenomeSeq and EdgeR) at FDR < 0.1, or identified as taxa discriminating between PwMS and HC by LEfSe at p-value <0.5 and LDA >1.8 in the Jewish cohort. Marked in grey – differential taxa identified uniquely in the Jewish cohort.
Abbreviations: FC- fold change, FDR- false discovery rate, HC- healthy controls, LDA - Linear Discriminant Analysis, ns- not significant

**Table S1B Taxa with differential relative abundance between PwMS and HC in the Arab cohort**

| Differentially abundant taxa | Differential abundance methods | | | | | | | |
|---|---|---|---|---|---|---|---|---|
| | EdgeR | | DeSeq2 | | MetagenomeSeq | LefSe | | Highest in |
| | FDR | FC | FDR | FC | FDR | P value | LDA | |
| **Order** | | | | | | | | |
| o__Coriobacteriales | ns | - | ns | - | ns | 0.0499 | 4.83 | MS |
| o__Fusobacteriales | $1.79^{-11}$ | 0.007 | ns | - | 0.0005 | ns | - | HC |
| **Family** | | | | | | | | |
| f__Clostridium_sp__K4410_MGS_306 | $2.04^{-9}$ | 0.03 | 0.045 | 0.02 | $6.57^{-5}$ | ns | - | HC |
| f__Coriobacteriaceae | ns | - | ns | - | ns | 0.024 | 4.84 | MS |
| f__Eggerthellaceae | ns | - | ns | - | ns | 0.036 | -4.07 | HC |
| f__Enterobacteriaceae | 0.002 | 0.13 | 0.045 | 0.18 | ns | ns | - | HC |
| f__Lactobacillaceae | ns | - | ns | - | ns | 0.042 | 4.54 | MS |
| f__Peptostreptococcaceae | ns | - | 0.0445 | 2.68 | 0.0968 | 0.014 | 4.65 | MS |
| f__Streptococcaceae | 0.0020 | 0.2 | 0.0445 | 0.26 | ns | ns | - | HC |
| **Genus** | | | | | | | | |
| g__Asteroleplasma | 0.0020 | 0.21 | ns | - | 0.0708 | ns | - | HC |
| g__Azospirillum_sp__47_25 | $1.52^{-7}$ | 0.04 | ns | - | 0.0241 | ns | - | HC |
| g__Clostridium_sp__K4410_MGS_306 | $6.50^{-9}$ | 0.03 | ns | - | $3.01^{-6}$ | NS | - | HC |
| g__Eubacterium_ventriosum_group | ns | - | ns | - | 0.003 | 0.023 | 3.81 | MS |
| g__Collinsella | ns | - | ns | - | 0.057 | 0.026 | 4.84 | MS |

| | | | | | | | | |
|---|---|---|---|---|---|---|---|---|
| g__Lactobacillus | ns | - | ns | - | ns | 0.042 | 4.54 | MS |
| g__Mailhella | $1.60^{-5}$ | 0.16 | ns | - | 0.080 | NS | - | HC |
| g__Mitsuokella | 0.0099 | 18.2 | ns | - | 0.024 | NS | - | MS |
| g__Moryella | ns | - | ns | - | NS | 0.043 | 2.73 | MS |
| g__Romboutsia | ns | - | ns | - | 0.0399 | 0.013 | 4.63 | MS |
| g__Roseburia | ns | - | ns | - | ns | 0.028 | 4.47 | MS |
| g__Ruminococcus_gnavus_group | 0.036 | 3.82 | 0.072 | 6.46 | ns | 0.014 | 2.95 | MS |
| g__Sarcina | 0.028 | 11.9 | ns | - | 0.028 | ns | - | MS |
| g__Tyzzerella_4 | $1.79^{-6}$ | 0.07 | ns | - | 0.028 | ns | - | HC |
| **Species** | | | | | | | | |
| s__Azospirillum_sp__47_25 | $1.54^{-7}$ | 0.05 | ns | - | 0.069 | ns | - | HC |
| s__Bacteroides_sp__3_2_5 | ns | - | 0.0002 | 73.4 | ns | 0.009 | 3.32 | MS |
| s__Clostridium_sp__K4410_MGS_306 | $1.79^{-9}$ | 0.03 | 0.064 | 0.03 | 0.0001 | ns | - | HC |
| s__Ruminococcus_gnavus_CC55_001C | 0.031 | 5.88 | 0.064 | 24.9 | 0.038 | 0.013 | 2.81 | HC |
| s__Ruminococcus_sp__UNK_MGS_30 | 0.022 | 12.0 | 0.065 | 18.2 | ns | 0.024 | 3.69 | HC |

Taxa with differential relative abundance in at least two out of three tests (DESeq2, MetagenomeSeq and EdgeR) at FDR < 0.1, or identified as taxa discriminating between PwMS and HC by LEfSe at p-value <0.5 and LDA >1.8 in the Arab cohort. Marked in grey – differential taxa identified uniquely in the Arab cohort.

Abbreviations: FC- fold change, FDR- false discovery rate, HC- healthy controls, LDA - Linear Discriminant Analysis, ns- not significant
