# Supplementary material for: Multiple Sclerosis-Associated Gut Microbiome in the Israeli Diverse Populations: Associations with Ethnicity, Gender, Disability Status, Vitamin D Levels, and Mediterranean Diet
Source: Int J Mol Sci. 2023 Oct 9;24(19):15024. doi: 10.3390/ijms241915024 (PMC10573818; doi:10.3390/ijms241915024)
Supplement: Supplementary file 1 [file ijms-24-15024-s001.zip › Table S2.pdf]

| Table S2 Comparison of nutrient intake between PwMS and HC | | | |
|---|---|---|---|
| **Nutrients** | MS (N=57) | Healthy Controls (N=39) | p-value |
| **Protein ± SE** | 100.7 ± 6.5 | 106.3 ± 11.2 | 0.45 |
| **Total fat (g) ± SE** | 88.6 ± 5.5 | 91.5 ± 9.8 | 0.63 |
| **Carbohydrates (g) ± SE** | 242.7 ± 16.4 | 246.0 ± 25.2 | 0.62 |
| **Food energy (Kcal) ± SE** | 2236.5 ± 139.6 | 2307.4 ± 234.8 | 0.548 |
| **Alcohol (g) ± SE** | 2.46 ± 0.8 | 3.52 ± 0.87 | **0.032** |
| **Moisture (g) ± SE** | 3347.4 ± 160.4 | 3547.0 ± 277.7 | 0.99 |
| **Total dietary fiber (g) ± SE** | 30.08 ± 2.39 | 30.23 ± 2.93 | 0.97 |
| **Calcium (mg) ± SE** | 1135.9 ± 69.6 | 1334.6 ± 215.9 | 0.25 |
| **Iron (mg) ± SE** | 12.93 ± 0.91 | 13.02 ± 1.19 | 0.97 |
| **Magnesium (mg) ± SE** | 509.1 ± 31.9 | 556.8 ± 50.3 | 0.64 |
| **Phosphorus (mg) ± SE** | 1653.6 ± 94.8 | 1817.7 ± 222.0 | 0.37 |
| **Potassium (mg) ± SE** | 4173.1 ± 279.2 | 4397.9 ± 415.7 | 0.91 |
| **Sodium (mg) ± SE** | 3939.0 ± 270.4 | 4124.3 ± 467.5 | 0.58 |
| **Zinc (mg) ± SE** | 11.9 ± 0.77 | 12.5 ± 1.31 | 0.59 |
| **Copper (mg) ± SE** | 1.99 ± 0.14 | 2.08 ± 0.23 | 0.94 |
| **Vitamin A iu (IU) ± SE** | 2519.6 ± 244.3 | 3050.3 ± 565.8 | 0.64 |
| **Vitamin A re (µg) ± SE** | 1011.0 ± 70.5 | 1207.0 ± 164.9 | 0.89 |
| **Carotene (µg) ± SE** | 6502.0 ± 629.2 | 6757.8 ± 708.9 | 0.59 |
| **Vitamin E (mg) ± SE** | 12.41 ± 0.97 | 12.45 ± 1.17 | 0.66 |
| **Vitamin C (mg) ± SE** | 218.9 ± 24.0 | 220.5 ± 22.8 | 0.50 |
| **Thiamin (mg) ± SE** | 1.39 ± 0.1 | 1.42 ± 0.15 | 0.76 |
| **Riboflavin (mg) ± SE** | 2.28 ± 0.14 | 2.72 ± 0.33 | 0.92 |
| **Niacin (mg) ± SE** | 28.56 ± 1.95 | 30.1 ± 2.46 | 0.67 |
| **Vitamin b6 (mg) ± SE** | 2.65 ± 0.18 | 2.69 ± 0.24 | 0.94 |
| **Folate (µg) ± SE** | 407.3 ± 32.0 | 430.6 ± 44.4 | 0.66 |
| **Vitamin b12 (µg) ± SE** | 5.44 ± 0.38 | 7.01 ± 1.08 | 0.93 |
| **Cholesterol (mg)± SE** | 344.3 ± 26.2 | 379.0 ± 41.6 | 0.86 |
| **Satured fat (g) ± SE** | 28.51 ± 1.77 | 30.97 ± 4.04 | 0.39 |
| **Butyric (g) ± SE** | 0.68 ± 0.05 | 0.9 ± 0.17 | 0.61 |
| **Caproic (g) ± SE** | 0.39 ± 0.03 | 0.5 ± 0.09 | 0.67 |
| **Caprylic (g) ± SE** | 0.33 ± 0.03 | 0.42 ± 0.08 | 0.78 |
| **Capric (g) ± SE** | 0.59 ± 0.04 | 0.76 ± 0.14 | 0.42 |

| | | | |
|---|---|---|---|
| **Lauric (g) ± SE** | 0.68 ± 0.05 | 0.87 ± 0.16 | 0.61 |
| **Myristic (g) ± SE** | 2.51 ± 0.18 | 3.25 ± 0.57 | 0.53 |
| **Palmitic (g) ± SE** | 13.91 ± 0.82 | 15.28 ± 1.88 | 0.50 |
| **Stearic (g) ± SE** | 5.94 ± 0.36 | 6.41 ± 0.82 | 0.33 |
| **Oleic (g) ± SE** | 31.78 ± 2 | 32.61 ± 3.31 | 0.79 |
| **Linoleic (g) ± SE** | 17.06 ± 1.27 | 15.99 ± 1.64 | 0.59 |
| **Linolenic (g) ± SE** | 2.69 ± 0.19 | 2.66 ± 0.31 | 0.47 |
| **Arachidonic (g) ± SE** | 0.17 ± 0.02 | 0.18 ± 0.02 | 0.84 |
| **Docosahexanoic (g) ± SE** | 0.12 ± 0.01 | 0.13 ± 0.01 | 0.31 |
| **Palmitoleic (g) ± SE** | 1.17 ± 0.08 | 1.29 ± 0.15 | 0.68 |
| **Parinaric (g) ± SE** | 0.03 ± 0 | 0.03 ± 0 | 0.38 |
| **Gadoleic (g) ± SE** | 0.38 ± 0.03 | 0.39 ± 0.04 | 0.98 |
| **Eicosapentaenoic (g) ± SE** | 0.05 ± 0.01 | 0.05 ± 0.01 | 0.37 |
| **Erucic (g) ± SE** | 0.07 ± 0.01 | 0.07 ± 0.01 | 0.32 |
| **Docosapentaenoic (g) ± SE** | 0.03 ± 0 | 0.03 ± 0 | 0.64 |
| **Mono unsatured fat (g) ± SE** | 35.02 ± 2.25 | 35.77 ± 3.61 | 0.75 |
| **Poly unsatured fat (g) ± SE** | 20.08 ± 1.44 | 19.06 ± 1.97 | 0.53 |
| **Vitamin d ± SE** | 7.78 ± 0.62 | 8.96 ± 1.38 | 0.68 |
| **Total sugars (g) ± SE** | 109.8 ± 8.4 | 116.8 ± 12.3 | 0.85 |
| **Trans fatty acids (g) ± SE** | 0.18 ± 0.02 | 0.23 ± 0.04 | 0.57 |
| **Isoleucine (g)± SE** | 4.13 ± 0.25 | 4.49 ± 0.51 | 0.38 |
| **Leucine (g) ± SE** | 7.26 ± 0.44 | 7.98 ± 0.93 | 0.38 |
| **Valine (g) ± SE** | 4.87 ± 0.3 | 5.37 ± 0.64 | 0.34 |
| **Lysine (g) ± SE** | 6.49 ± 0.42 | 7.15 ± 0.83 | 0.42 |
| **Threonine (g) ± SE** | 3.67 ± 0.23 | 3.96 ± 0.43 | 0.42 |
| **Methionine (g) ± SE** | 2.11 ± 0.13 | 2.28 ± 0.26 | 0.41 |
| **Phenylalanine (g) ± SE** | 4.28 ± 0.27 | 4.58 ± 0.52 | 0.40 |
| **Tryptophan (g) ± SE** | 1 ± 0.06 | 1.07 ± 0.12 | 0.46 |
| **Histidine (g) ± SE** | 2.55 ± 0.16 | 2.76 ± 0.3 | 0.40 |
| **Tyrosine (g) ± SE** | 3.46 ± 0.23 | 3.77 ± 0.46 | 0.35 |
| **Arginine (g)± SE** | 5.07 ± 0.33 | 5.22 ± 0.5 | 0.49 |
| **Cystine (g) ± SE** | 1.17 ± 0.08 | 1.19 ± 0.12 | 0.53 |
| **Serine (g) ± SE** | 4.35 ± 0.26 | 4.8 ± 0.56 | 0.39 |
| **Vitamin K ± SE** | 238 ± 21.8 | 207.7 ± 19.5 | 0.55 |

| | | | |
|---|---|---|---|
| **Pantothenic acid (mg) ± SE** | 7.96 ± 0.51 | 8.55 ± 1 | 0.49 |
| **Iodine ± SE** | 0 ± 0 | 0 ± 0 | 1.0 |
| **Selenium (µg) ± SE** | 132.9 ± 9.9 | 141.9 ± 14.3 | 0.89 |
| **Sugar alcohols (g) ± SE** | 0.71 ± 0.3 | 0.35 ± 0.09 | 0.75 |
| **Choline (mg) ± SE** | 451.9 ± 29.3 | 486.9 ± 48.9 | 0.83 |
| **Biotin (µg) ± SE** | 0.94 ± 0.17 | 0.89 ± 0.26 | 0.23 |
| **Manganese (mg) ± SE** | 3.84 ± 0.26 | 3.96 ± 0.35 | 0.70 |
| **Fructose (g) ± SE** | 21.39 ± 2.65 | 23.02 ± 2.67 | 0.41 |
| **Animal protein grams (g) ± SE** | 64.69 ± 4.76 | 71.43 ± 8.71 | 0.53 |

Analysis of FFQ providing data on the daily energy intake and of 74 nutrients. The daily intake was compared between PwMS and healthy controls (HC) by Mann-Whitney U test.
Abbreviations: g-gram, SE- standard error.
