# Supplementary material for: Multiple Sclerosis-Associated Gut Microbiome in the Israeli Diverse Populations: Associations with Ethnicity, Gender, Disability Status, Vitamin D Levels, and Mediterranean Diet
Source: Int J Mol Sci. 2023 Oct 9;24(19):15024. doi: 10.3390/ijms241915024 (PMC10573818; doi:10.3390/ijms241915024)
Supplement: Supplementary file 1 [file ijms-24-15024-s001.zip › Table S4.pdf]

Table S4 Correlations between MS-differentially abundant taxa and nutrients, which support a “repair” of altered microbiota in PwMS

Nutrient	Differentially abundant OTU (MS/H)	Enriched/ reduced in PwMS	p-value (Rho)	Nutrients	Differentially abundant OTU (MS/H)	Enriched/ reduced in PwMS	p-value (Rho)
**Vitamin B12**	Eggerthella (g)	Enriched	0.047 (-0.203)	**Poly Unsaturated fat**	Eggerthella (g)	Enriched	0.018 (-0.241)
**Choline**	Eggerthella (g)	Enriched	0.017 (-0.244)		Flavonifractor (g)	Enriched	0.022 (-0.233)
**Vitamin E**	Flavonifractor (g)	Enriched	0.047 (-0.203)	**Trans fatty acids**	Ruminococcus gnavus CC55_001C (s)	Enriched	0.044 (0.206)
**Folate**	Flavonifractor (g)	Enriched	0.034 (-0.217)		Ruminococcaceae UCG-013 (g)	Reduced	0.049 (-0.202)
**Vitamin A**	Mitsuokella (g)	Enriched	0.050 (-0.201)	**Saturated fat**	Ruminococcaceae UCG-013 (g)	Reduced	0.037 (-0.213)
**Carotene**	Mitsuokella (g)	Enriched	0.029 (-0.223)		uncultured Clostridium sp. (s)	Reduced	0.039 (-0.211)
	Negativibacillus (g)	Enriched	0.022 (-0.234)	**Capric**	Bacteroides timonensis (s)	Reduced	0.041 (-0.209)
**Vitamin K**	Negativibacillus (g)	Enriched	0.044 (-0.206)	**Palmitic**	Bacteroides timonensis (s)	Reduced	0.046 (-0.204)
**Vitamin C**	Lachnospiraceae UCG-004 (g)	Reduced	0.012 (0.257)	**Magnesium**	Peptostreptococcaceae (f)	Enriched	0.038 (-0.205)
**Riboflavin**	Lachnospiraceae UCG-004 (g)	Reduced	0.046 (0.204)		Romboutsia (g)	Enriched	0.024 (-0.230)
**Vitamin D**	Lachnospiraceae UCG-004 (g)	Reduced	0.040 (0.201)	**Copper**	Flavonifractor (g)	Enriched	0.022 (-0.234)
**Linoleic (omega 6)**	Eggerthella (g)	Enriched	0.024 (-0.230)		Negativibacillus (g)	Enriched	0.048 (-0.202)
	Flavonifractor (g)	Enriched	0.031 (-0.220)	**Manganese**	Flavonifractor (g)	Enriched	0.015 (-0.249)
**Arachidonic (omega 6)**	Eggerthella (g)	Enriched	0.014 (-0.250)	**Calcium**	Lachnospiraceae UCG-004 (g)	Reduced	0.041 (0.209)
**Docosahexanoic (DHA) (omega 3)**	Eggerthella (g)	Enriched	0.025 (-0.229)	**Cysteine**	Eggerthella (g)	Enriched	0.045 (-0.205)
**Parinaric**	Eggerthella u(g)	Enriched	0.028 (-0.224)	**Dietary fibers (g)**	Flavonifractor (g)	Enriched	0.044 (-0.207)
	Mitsuokella (g)	Enriched	0.047 (-0.203)	**Fructose**	Clostridium sp. K4410.MGS-306 (f)	Reduced	0.041 (-0.209)
**Gadoleic (omega 9)**	Eggerthella (g)	Enriched	0.015 (-0.247)		Escherichia-Shigella (g)	Reduced	0.046 (-0.204)
**Eicosapentaenoic (EPA) (omega 3)**	Eggerthella (g)	Enriched	0.046 (-0.204)		Clostridium sp. K4410.MGS-306 (s)	Reduced	0.041 (-0.209)
**Erucic (omega 9)**	Eggerthella (g)	Enriched	0.017 (-0.244)		Escherichia coli (s)	Reduced	0.035 (-0.215)
**Docosapentaenoic (DPA)**	Eggerthella (g)	Enriched	0.024 (-0.230)				
	Mitsuokella (g)	Enriched	0.033 (-0.218)				

Associations between MS- differentially abundant OTUs and nutrients were assessed by Spearman correlation (shown in Supplemental Table 3). Significant correlations which direction supports “a repair” of the altered abundance in PwMS are included in the table.
Abbreviations: F-family, g-genus, s-specie.
