# Supplementary material for: Multiple Sclerosis-Associated Gut Microbiome in the Israeli Diverse Populations: Associations with Ethnicity, Gender, Disability Status, Vitamin D Levels, and Mediterranean Diet
Source: Int J Mol Sci. 2023 Oct 9;24(19):15024. doi: 10.3390/ijms241915024 (PMC10573818; doi:10.3390/ijms241915024)
Supplement: Supplementary file 1 [file ijms-24-15024-s001.zip › Table S5.pdf]

Table S5 Enriched KEGG pathway and network mapping of significantly differential KOs between MS and HC

KEGG pathway	total	Expected	Hits	FDR
Biosynthesis of amino acids	223	11.2	50	1.55E-19
2-Oxocarboxylic acid metabolism	67	3.35	12	0.006
Peptidoglycan biosynthesis	17	0.851	6	0.006
Pyrimidine metabolism	85	4.26	12	0.035
Alanine, aspartate and glutamate metabolism	65	3.25	10	0.039
One carbon pool by folate	27	1.35	6	0.041
D-Glutamine and D-glutamate metabolism	6	0.3	3	0.041
Geraniol degradation	6	0.3	3	0.041
Histidine metabolism	38	1.9	7	0.041

Significantly enriched KEGG pathway and network mapping of 485 KOs, predicted by Tax4Fun2, that differ significantly between PwMS and healthy controls (HC) at FDR<0.1 (Kruskal-Wallis).
