# Supplementary figures and images for: Multiple Sclerosis-Associated Gut Microbiome in the Israeli Diverse Populations: Associations with Ethnicity, Gender, Disability Status, Vitamin D Levels, and Mediterranean Diet
Source: Int J Mol Sci. 2023 Oct 9;24(19):15024. doi: 10.3390/ijms241915024 (PMC10573818; doi:10.3390/ijms241915024)

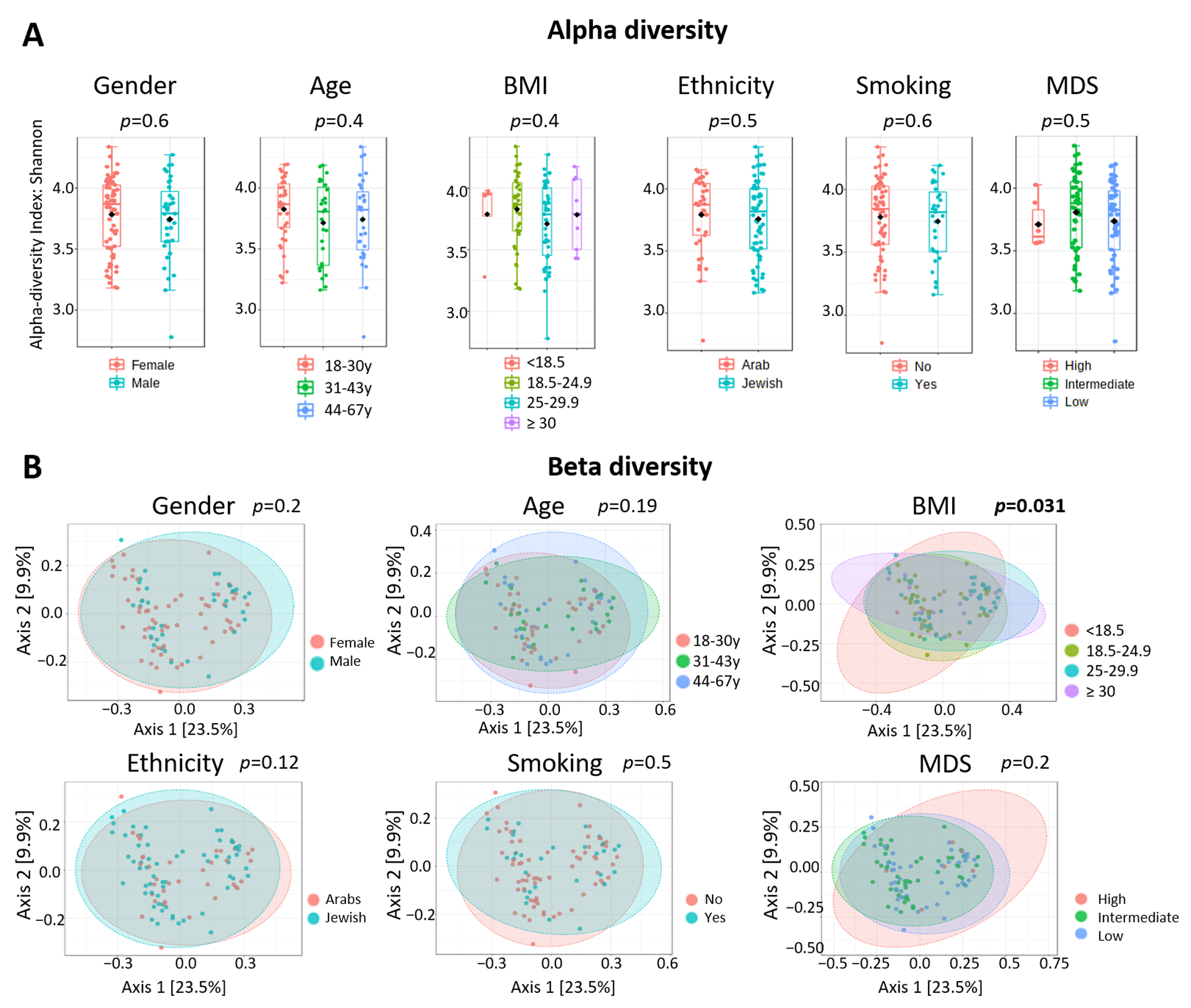

Supplement: Supplementary file 1 [file ijms-24-15024-s001.zip › Figure S1.tif]
